# Progress in Topical and Transdermal Drug Delivery Research—Focus on Nanoformulations

**DOI:** 10.3390/pharmaceutics16060817

**Published:** 2024-06-16

**Authors:** Dominique Lunter, Victoria Klang, Adina Eichner, Sanela M. Savic, Snezana Savic, Guoping Lian, Franciska Erdő

**Affiliations:** 1 Department of Pharmaceutical Technology, Eberhard-Karls-Universität Tübingen, 72074 Tübingen, Germany; dominique.lunter@uni-tuebingen.de; 2 Department of Pharmaceutical Sciences, University of Vienna, 1010 Vienna, Austria; victoria.klang@univie.ac.at; 3 Department of Dermatology and Venereology, Martin Luther University Halle-Wittenberg, 06108 Halle, Germany; adina.eichner@medizin.uni-halle.de; 4 Institute of Applied Dermatopharmacy, Martin Luther University Halle-Wittenberg (IADP) e.V., 06108 Halle, Germany; 5 Faculty of Technology in Leskovac, University of Niš, 16000 Leskovac, Serbia; saneladjordjevic87@gmail.com; 6 R&D Sector, DCP Hemigal, 16000 Leskovac, Serbia; 7 Department of Pharmaceutical Technology and Cosmetology, Faculty of Pharmacy, University of Belgrade, 11000 Belgrade, Serbia; snezana.savic@pharmacy.bg.ac.rs; 8 Department of Chemical and Process Engineering, University of Surrey, Guildford GU2 7XH, UK; g.lian@surrey.ac.uk; 9 Unilever R&D Colworth, Sharnbrook, Bedford MK44 1LQ, UK; 10 Faculty of Information Technology and Bionics, Pázmány Péter Catholic University, 1083 Budapest, Hungary

**Keywords:** skin structure and function, topical administration, transdermal delivery, drug (nano)formulation, nanoparticulate delivery systems, skin penetration, Franz diffusion cell, skin-on-a-chip microfluidic devices, confocal Raman spectroscopy, PBPK modeling of skin penetration

## Abstract

Skin is the largest organ and a multifunctional interface between the body and its environment. It acts as a barrier against cold, heat, injuries, infections, chemicals, radiations or other exogeneous factors, and it is also known as the mirror of the soul. The skin is involved in body temperature regulation by the storage of fat and water. It is an interesting tissue in regard to the local and transdermal application of active ingredients for prevention or treatment of pathological conditions. Topical and transdermal delivery is an emerging route of drug and cosmetic administration. It is beneficial for avoiding side effects and rapid metabolism. Many pharmaceutical, technological and cosmetic innovations have been described and patented recently in the field. In this review, the main features of skin morphology and physiology are presented and are being followed by the description of classical and novel nanoparticulate dermal and transdermal drug formulations. The biophysical aspects of the penetration of drugs and cosmetics into or across the dermal barrier and their investigation in diffusion chambers, skin-on-a-chip devices, high-throughput measuring systems or with advanced analytical techniques are also shown. The current knowledge about mathematical modeling of skin penetration and the future perspectives are briefly discussed in the end, all also involving nanoparticulated systems.

## 1. Introduction

Currently, in addition to traditional and parenteral oral dosage forms, alternative drug administration methods, including topical (or also called dermal) and transdermal delivery systems of active pharmaceutical ingredients (APIs), are becoming more and more important. While topical/dermal formulations are aimed to achieve local effects on the skin surface/superficial layers (e.g., epidermis—topical delivery) and in the underlying tissues (e.g., dermis—dermal delivery; muscle or joint), transdermal formulations are designed to reach systemic circulation by penetrating the skin layers to achieve systemic effects [1]. Therefore, the skin/transdermal pathway is not only important for dermatological therapy, as other organs (e.g., joints, central nervous system, hormone therapy, or pain relief, etc.) can also be the therapeutic targets. In addition, most of the preparations developed for the purposes of cosmetology are also applied to the skin or skin appendages (hair, nails). However, due to the specific functionalities of its morphology, human skin poses an efficient mechanical barrier against the ingress of substances that come into contact with the skin surface. Many active ingredients are difficult to absorb via the skin barrier [2], so they can only be effectively delivered through the skin in appropriate formulations and with penetration-enhancing techniques.

Navigating through the landscape of recent scientific papers, patent applications and clinical trials, it is evident that progress has been extensively made in the development and application of various modern, innovative, topical and transdermal delivery systems and methods for enhancing skin penetration and permeation. The present review deals with the different aspects of drug and cosmetic penetration into and/or across the skin barrier, involving versatile techniques used for characterization of skin penetration as well as mathematical modeling for penetration prediction. An overview of conventional topical drug delivery systems and, particularly, of advanced, nanoparticle-based carriers for topical and transdermal drug delivery is also presented through an extensive survey of recently reported research studies, clinical trials and patent applications in the field.

## 2. Drug Delivery through the skin

### 2.1. Structure of the Skin and Its Barrier Function

**Figure 1:**
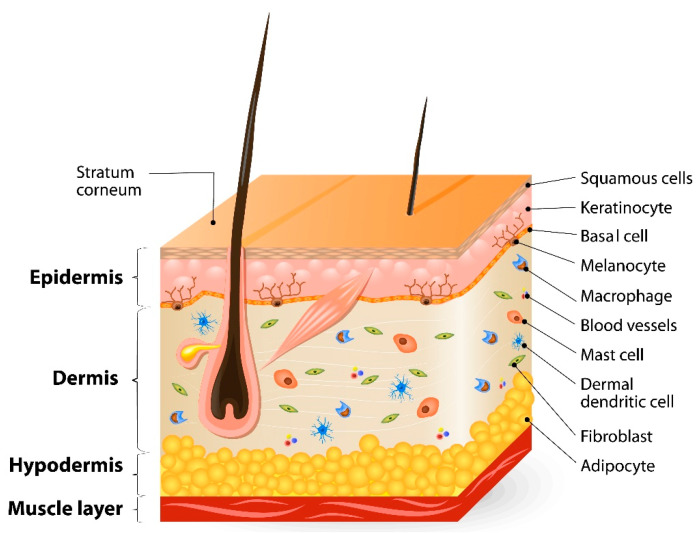
Main cellular elements of the human skin.

How is the skin barrier structured? This knowledge is of high importance in regard to finding strategies to overcome the barrier for therapeutic reasons and to investigate the penetration behavior of APIs. The skin features a layer-based structure which is constantly renewing itself. Figure 1 presents a scheme of the skin cross section, which is differentiated into its main structures of the epidermis and dermis.

The largest structure of human skin is the dermis, in which hair follicles and their erecting muscles, perspiratory and sebaceous glands, nerves, small blood and lymph vessels are located. For the skin immune function, physiologically important macrophages, mast cells and dermal dendritic cells are also found in this layer. Its strength, as well as its flexibility, is induced by fibroblasts and structure proteins like collagen and elastin bundles [3]. The epidermis can be divided into a viable and a non-viable section. The viable section beneath the squamous epidermis cells is composed of basal cells and melanocytes. Furthermore, the outermost layer of the non-viable section is the so-called stratum corneum (SC), a very thin but highly defined heterogenous microstructure with cornified cells (corneocytes) which are surrounded by a complex lamellar lipid matrix—comparable to a wall—where bricks are embedded in mortar. This is what the brick mortar model was named after [4]. The corneocytes themselves are surrounded by covalently bound lipid lamellae, which form the cornified lipid envelope (CLE). Thus, the corneocytes are connected to each other by so-called corneodesmosomes, meaning cell–cell contacts, where up to 500 are described per corneocyte. All SC components and their interplay have been the focus of international research groups in recent decades, and this interest is still ongoing. A central point of interest has been the exact qualitative and quantitative composition of the SC lipid matrix, where ceramides, cholesterol, cholesterol sulphate, and free fatty acids were defined as main components [5]. They were used to investigate skin lipid models to understand their impact on the SC barrier function [6]. This is of high relevance, as either pathogenic event occurs as a change in the lipid composition followed by a disturbed skin barrier [7]. Interruptions or defects within the SC lipid matrix and changes in lipid conformation render the SC more permeable for both lipophilic and hydrophilic compounds [8]. Dehydration of the SC, which, in a diseased state, is often related to decreased content of the natural moisturizing factor, causes increased scaling and weakened barrier function [9], while increased water content causes swelling and facilitates ingress, especially of polar permeants [10]. Therefore, the composition, structure and organization of SC lipids play essential roles in maintaining normal skin hydration and physiological function [11].

Furthermore, the SC barrier function is strongly dependent on intact physiological conditions. In practice, this also involves an intact hydrolipidic film at the skin surface with a balanced skin microbiome [12]. Maintaining acidic physiological pH to ensure adequate enzymatic activity of both endogenous and microbial enzymes ensures formation of the SC compounds to maintain homeostasis.

### 2.2. Penetration Pathways through the Stratum Corneum (SC)

In regard to transdermal drug penetration, beside the SC, which makes the primary barrier, the viable epidermis is also considered rate-limiting to a certain extent, while the underlying dermis is considered a significant limitation mostly for highly lipophilic permeants [13]. The available penetration pathways are described as axillary via the SC and alongside the hair follicle shafts and sweat ducts, where the latter can also contribute significantly to overall absorption and permeation through the skin [14]. However, as these skin appendages comprise a limited area, they are assumed to play their significant role only under specific circumstances [15]. The most significant contribution to steady state drug flux across the skin is assumed to be achieved through intercellular transport, i.e., penetration along the SC lipid matrix [8]. Since the SC lipid matrix offers a continuous phase for drug transport and—due to its internal structure—sufficient solubility for moderately hydrophilic to lipophilic drugs, it is generally recognized that most uncharged molecules with a small molecular mass (<500 Da) will penetrate along this pathway [13]. In addition, drug penetration through the transcellular pathway directly across corneocytes and intercellular lipid space is possible. However, due to the permanent change in hydrophilic/lipophilic regions, this pathway plays a subordinate role regarding its relative contribution to steady state flux. But, solute diffusion across the SC barrier can take place via the trans-corneocyte (corneodesmosomal) pathway alongside the surface of the corneocytes as well, crossing the lipid matrix via corneodesmosomes [16].

### 2.3. Skin Models with Different Complexity

Evaluation and optimization of topical and transdermal formulations is another significant challenge. Today, researchers are increasingly moving from preclinical animal models to in vitro early phase studies and human testing. Testing cosmetics on experimental animals is prohibited; however, drugs can still be assessed on in vivo animal models. Animal experiments are of great importance in modeling various dermatological diseases, discovering their pathomechanisms, developing different therapeutic strategies, and identifying targets. Moreover, devices with a miniaturized size, better simulating physiological conditions [17], and higher dynamicexamination reveal the absorption of drugs and active substances ex vivo through skin tissue samples or skin cell cultures. There are many types of these systems that can be used to model both healthy and diseased states. Significant progress has been made recently in the development of various skin substitutes. Thus, in addition to two-dimensional (2D) and three-dimensional (3D) in vitro cell culture assays [18], many researchers are also working on the production of 3D bioprinted artificial tissues [19]. The combination of one-, two- and three-component tissues (keratinocytes, fibroblasts and melanocytes) with vascular and immune elements (endothelial cells, macrophages, etc.) represents the most relevant approach at the moment. Further efforts have been made to integrate skin appendages into artificial skins. If these studies do not produce extensive results, model calculations are a significant contribution in estimating the skin absorption of drugs, as they can predict with a very good approximation the absorption of active substances under different conditions.

The following review provides a comprehensive overview of the available methods, techniques and their various advantages and disadvantages with regard to local and transdermal drug diffusion approaches.

## 3. Skin Penetration

### 3.1. Biophysical Aspects of the Skin Penetration and Permeation

Skin permeation of drugs through the SC depends on the physicochemical properties of the applied substance, vehicle properties, and skin condition [10]. In dermal drug delivery, drug penetration into and distribution within the epidermis and dermis is the main target. Drug penetration into surrounding adipose and muscle tissue may be observed depending on drug properties. Successful design of a transdermal drug delivery system (TDDS) involves further uptake into the vascular system and distribution within the systemic blood circulation. Considering the steady-state flux, as defined by Barry [20], the most important factors governing drug permeation can be reduced to:

(1) $$J=\frac{D\;c_{0}\;K}{h}$$

The drug mass flux in steady-state conditions J is determined by the diffusion coefficient D of the drug, the constant donor drug concentration $c_{0}$ and the partition coefficient K between the membrane and acceptor solution. The higher these factors are, the higher the mass flux will be. An inverse correlation is found for the membrane thickness h, where larger values will cause lower mass flux per unit of time and area. Consequently, desired properties to optimize drug flux across the SC are as follows [13,20]: (I) low molecular mass, ideally below 500 Da, and high D; (II) moderate lipophilicity to achieve adequate solubility in both oil and water, thus achieving high thermodynamic activity through a high membrane concentration gradient (III); balanced *K* to achieve good partitioning into the membrane, but also clearance towards the viable tissue: hence, intermediate partition coefficients (log *K* octanol/water of 1–3) are to be preferred; (IV) a low melting point, ensuring solubility at room temperature.

If these properties are not sufficiently met to ensure an adequate drug flux across the SC, different enhancement strategies can be followed to meet the therapeutic goal. For transdermal delivery, the free base or acid form of small lipophilic drugs is usually preferred over their salt forms [21]. For the increasing number of modern biotech drugs, such as peptides, proteins and bionucleotides, the following additional challenges must be faced: large molecular weight, their polar nature and/or charged or even zwitterionic groups that hinder successful delivery across the SC by passive diffusion and render enhancement strategies a necessity [21].

### 3.2. Enhancement of Skin Penetration/Permeation: Chemical and Physical Strategies

To achieve adequate dermal or transdermal drug concentrations for therapeutic success, many active pharmaceutical ingredients require the use of enhancement strategies that temporarily lower SC barrier function. Two main options exist: the use of chemical permeation enhancers, i.e., passive methods that affect SC barrier function through application of specific chemical agents or vehicles, and the use of physical enhancement methods, i.e., active methods that involve an energy input, such as sonophoresis, iontophoresis and magnetophoresis, or disruption of the SC barrier through microneedling, microdermabrasion or tape stripping.

Chemical enhancers have been extensively investigated in past decades [22]. Water may increase drug penetration through swelling of the SC, which corresponds to the effect achieved by occlusion through lipophilic ointments or patches. Most chemical enhancers lower the barrier function of the SC lipid matrix through lipid extraction, intercalation or fluidization [23]. The mechanism of action of chemical enhancers is entirely dependent on their structural properties; effects have been described for dimethyl sulphoxide, azone, alcohols such as ethanol and propylene glycol, surfactants including phospholipids and fatty acids, such as oleic acid [24] and terpenes [25]. Cell-penetrating peptides are among the most recent approaches [26]. Vehicle strategies to promote drug penetration into the skin involve adaptation of the vehicle to increase mass flux across the SC, e.g., by supersaturation, formation of solvent-rich microemulsions or use of ionic liquids. Various nanocarrier systems have been developed to achieve the enhanced skin penetration of actives, e.g., liposomes, lipid nanoparticles, carbon nanotubes or dendrimers. Apart from vehicle effects, drug modification is possible to improve penetration, e.g., by use of prodrugs [27].

Physical penetration enhancement can be achieved by mechanically disrupting the SC barrier. Currently proposed strategies include treatment with microneedles or dermarollers [28,29], elongated microparticles [29] and, more recently, STAR particles [30]. Reported disadvantages of such techniques include variability in penetration depth and lack of efficacy in some cases [29].

### 3.3. Penetration of Cosmetics vs. Therapeutic Formulations

As both therapeutic and cosmetic formulations have comparable excipients and formulae, they are available in the form of oil, ointment, emulsion, gel, lotion, aqueous solution, paste or even powder. The fact that cosmetic and pharmaceutically active formulations differ in terms of the effects they impart is primarily due to the different regulations to which both types of preparations are subject. Cosmetics, including their manufacturing, permitted and prohibited ingredients and labeling requirements, are regulated at the European level by Regulation (EC) No. 1223/2009 [31], which states that cosmetics may only care, cleanse, perfume or maintain a good condition. Pharmaceutically active preparations are subject to the European Directive 2001/83/EC [32], which, when transposed into national law, states that these preparations must have a pharmacological, metabolic or immunological effect on the human body in order to be a medicinal product. It is therefore clear from the mediating effects and interactions of the preparations with the human body to what extent cosmetics and medicinal products differ. Nevertheless, there are always borderline cases that run not least along the pharmacologically active concentration of an ingredient or are even to be defined as a substance-based medical device according to Regulation (EU) No. 2017/745 [33], which primarily conveys a medical purpose due to physical effects, e.g., pH regulation, isotonicity, cooling or UV protection. Numerous cosmetic formulations aim to cleanse the skin as rinse-off products or to perfume the skin as leave-on products. Cosmetics, furthermore, are primarily used for skin care, in particular for hydrating the skin or supplying skin-like substances such as ceramide subspecies, hyaluronic acid or vitamins in order to maintain or restore a good condition. However, following Regulation (EC) No. 1223/2009 [31], cosmetics are only allowed to penetrate into the skin, whereby permeation and thus systemic bioavailability should always be excluded to avoid negative side effects.

Pharmaceutical-active formulations, on the other hand, are built around the active ingredient and aim to maximize its bioavailability. Depending on the target compartment, the penetration depths of the active ingredients also differ, as follows: azoles as antimycotics act locally on or in the SC; glucocorticoids or calcineurin inhibitors for the treatment of, e.g., atopic dermatitis act primarily in the dermis; transdermal topical preparations, such as hormone gels, should reach the systemic circulation with correspondingly high and constant bioavailability.

## 4. Liquid and Semisolid Topical/Transdermal Formulations

### 4.1. Classical Topical Formulations

Despite extensive research on innovative formulations for dermal and transdermal use, most marketed formulations still fall within one of the classical topical formulation types. Therefore, the systematics of classical topical formulations will be described briefly in the following paragraphs. Classical topical formulations can be divided by (a) their viscosity into liquid or semisolid formulations; (b) by the number of phases, which may be one or two; and (c) by the site of action of the comprised drug, which may be the skin itself (dermal) or any part of the body after systemic absorption (transdermal). Table 1 gives an overview of the classification by the European Pharmacopoeia 11th edition (Ph. Eur. 11.0) [34]. Emulsions and creams contain a minimum of two phases, i.e., oil and water, wherein one phase is distributed within the other. Thereby, water-in-oil (W/O) or oil-in-water (O/W) formulations may be formed. Emulsifiers are added to enable emulsification and ensure physical stability of the emulsion during storage. They are classified by their hydrophilic-lipophilic balance (HLB) value. If the lipophilic part of the molecule dominates, values below 10 are achieved which relate to W/O-emulsifiers. Accordingly, values above 10 are obtained if the hydrophilic part dominates and describe O/W-emulsifiers. The oil component may be used to dissolve the API, to improve the skin feel, to improve the skin care properties or to deliver penetration enhancers (e.g., isopropyl myristate, oleic acid or essential oils).

Semisolid dosage forms for cutaneous use exhibit a yield point, which means that a certain (shear) force is required to make them flow. This behavior enables easy dosing and spreadability while ensuring that the formulation stays in place once applied to the skin.

Gels contain gelling agents that immobilize the liquid. In principle, three mechanisms of gel formation are described. Polymeric gel formers, such as cellulose ethers, may interact with each other by secondary valences (Van der Waals forces or hydrogen-bonding); surfactants form micelles, which, in cases of a suitably high concentration, lead to an increased viscosity and development of a yield point, and particles may form networks due to inter-particulate interactions and thereby immobilize the fluids between them. In hydrogels, electrostatic repulsion of, for example, deprotonated acids may also lead to entrapment of the liquid phase and gel formation. Here, the negatively charged -COO^−^ groups present within the polymer chains repulse each other, leading to swelling and subsequent entrapment of water. Hydrogels have a cooling effect and “light” skin feel, whereas oleogels are partially occlusive and may be perceived as greasy.

Hydrophilic ointments are scarce; most ointments are hydrophobic or water absorbing. In the latter case, the comprised emulsifier(s) and its/their HLB-value(s) determine the type of cream formed [34]. Some of the substances comprising the term emulsifier may rather be termed as constancy agents, like glycerol monostearate or cetylstearyl alcohol. Although a (very low) HLB value can be calculated, the substances are not good emulsifiers as they crystallize at room temperature. The benefit of their incorporation into ointments and creams rather is the formation of a liquid crystalline network inside the formulation which contributes to the formation of a yield point and thus to the semisolid nature of the formulation.

Classical creams are formed out of water-absorbing ointments by adding water. The HLB value of the emulsifier present in the water-absorbing ointment defines the type of cream that is obtained. In the classical picture of creams, they comprise two phases, water and oil. Today, creams are understood as tri-phasic systems as the emulsifiers form a separate, liquid crystalline phase together with constancy agents. The formed network provides the creams with their semisolid behavior [35].

Addition of larger amounts of finely dispersed solids, such as ZnO to creams or ointments leads to the formation of pastes.

Apart from these classical creams, more sophisticated formulations are developed that do not entirely fit the categories of the pharmacopoeia. Some recently approved products, researched from the EMA’s and FDA’s database, will be highlighted here. An example is Wynzora^®^ cream, which makes use of the PAD™ technology (PolyAphron Dispersion), where the oil droplets are stabilized due to encapsulation in a multimolecular structure formed by the emulsifiers [36], thereby providing such high stability to the individual droplets that the emulsifier content can be drastically reduced. This does not only make the formulation less irritative but also provides higher chemical stability to drugs that are prone to hydrolysis. Another example is Twyneo^®^, cream for treatment of acne, marketed in the US. It combines tretinoin and benzoyl peroxide in one formulation, although tretinoin is prone to oxidation and benzoyl peroxide is a strong oxidizing agent. To achieve this, the marketing authorization holder claims both drugs are encapsulated separately inside the cream so that tretinoin is chemically stable despite the presence of benzoyl peroxide [37]. Butylated hydroxytoluene as an antioxidant and citric acid as a pH adjustment agent surely help in keeping tretinoin stable.

As one can deduce from the last examples, and which will become even more obvious in the following chapters, new formulation categories are urgently needed to fit the innovative techniques that are currently being developed.

### 4.2. Film Forming Formulations

Film forming formulations were pioneered by Lunter in 2012 [38,39]. The formulations combine the advantages of semisolid formulations with those of patches. Like semisolid formulations, they are easy to spread on the skin and may be applied to skin areas of any size. After application, they form a flexible film on the skin which, like patches, contains a reservoir of the drug and releases it continuously; therefore, high substantivity and a sustained release can be achieved [40]. Film-forming formulations may be solutions or emulsions that contain film-forming polymers either in solution or dispersion, and mesoporous carriers may also be used to incorporate the drug [38,39,40]. Organic solvents like ethanol or isopropanol, as well as hydro-alcoholic solutions, are used to dissolve drugs and polymers [41].

Special aspects to be considered in the design of film forming formulations include the solubility of the drug in the solvent and/or polymer, the skin irritancy of the formulation as a whole, flexibility of the formed film, impact of the excipients on skin barrier function and environmental aspects, to name a few. For example, organic solvents evaporate quickly upon application to the skin and leave a film comprising the drug in amorphous dispersions. These are unstable, and precipitation of the drug into small particles may occur, which would then alter the release kinetics [35]. To circumvent negative impacts of organic solvents on the skin barrier, aqueous dispersions of non-water-soluble polymers may be used as an alternative to organic solutions. Sustained release polymers like Eudragit RS, NE may be used as film formers that guarantee sustained release of the incorporated drug, but they are not biodegradable and will enter the environment through wastewater [39,42]. Thus, natural polymers like levan have also been investigated, just like semi-synthetic polymers, e.g., cellulose ethers [43,44]. Both film forming solutions and emulsions may contain plasticisers to make the film more flexible and enable them to follow the normal movements of the human skin [38,39,43]. Emulsions may be used to dissolve non-water-soluble drugs in the oil phase and prevent precipitation after film formation. The oil phase may also serve as an emollient, which may be beneficial for therapy [44]. The patch-no-patch system follows a different approach to the previously described film forming formulations. The drug, plasticiser, optional penetration enhancer and polymer are dissolved in water, cast to a defined thickness and dried to give a thin film. Before application to the skin, the films are wetted by a small amount of water, which makes the polymer swell and become adhesive. This enables the patches to be applied to the skin without the necessity of adding additional adhesives [45].

Kapoor et al. developed film forming formulations comprising tazarotene as an active ingredient and polyamide-3 polymers as film formers [35]. Upon application to the skin, films were formed in which the active ingredient was molecularly dispersed, as shown by a decrease in the polymer-glass transition temperature. Release and penetration of the active substance from the films was ten-fold increased compared to a marketed conventional formulation [35]. This shows that the concept of using amorphous solid dispersions to enhance bioavailability may also be applied in topical dosage forms.

Han et al. developed film forming formulations containing acyclovir [46]. Using chitosan and polyvinyl pyrrolidone as film formers, combined with PEG 600 as a plasticiser and lactic acid as a humectant, they were able to form hydro-alcoholic gels which rapidly form films on the skin and inhibit excessive crystallization of the drug therein. They also found that high amounts of PEG 600 enhanced acyclovir penetration into the skin, which resulted in significantly increased delivery of acyclovir compared to a marketed conventional formulation [46].

Chamsai et al. developed chlorhexidine gluconate containing film forming formulations for the antiseptic treatment of wounds [47]. After evaluation of different polymers (Eudragit S, Eudragit L and polyvinyl alcohol), they found that the films generated from polyvinyl alcohol gave the best results as they not only showed satisfactory film properties but also inhibited the growth of *Staphylococcus aureus* and did not interfere with wound healing.

Seljak et al. developed nanocellulose-based film forming formulations containing betamethasone dipropionate for the treatment of atopic dermatitis [48]. Due to its low water solubility, they formulated the active as a self-micro-emulsifying drug delivery system which was subsequently incorporated into nanocellulose-based hydrogels containing additional natural polymers. The films were able to reduce the transepidermal water loss (TEWL) for a short time after application but were found not to be occlusive, as the TEWL returned to normal values after approx. 30 min. The type of nanocellulose used had a significant effect on drug release, where thicker and longer fibers formed bigger pores and thus showed faster release. Altogether, drug release from all tested films was found to be significantly prolonged, which was deemed beneficial for patient compliance [48].

These recent developments show that there still is an unbroken interest in film forming formulations for treatment of various diseases. The polymers used range from hydrophobic polyamides to hydrophilic natural polymers and must be chosen according to disease and drug. Penetration enhancement can be achieved when releasing the drug through amorphous dispersion, and sustained release can be achieved by the combination of a self-micro-emulsifying drug delivery system (SMEDDS) and hydrogels. Film-forming formulations thus represent a versatile formulation concept in topical drug therapy.

### 4.3. Advanced (Trans)dermal Formulations

**Figure 2:**
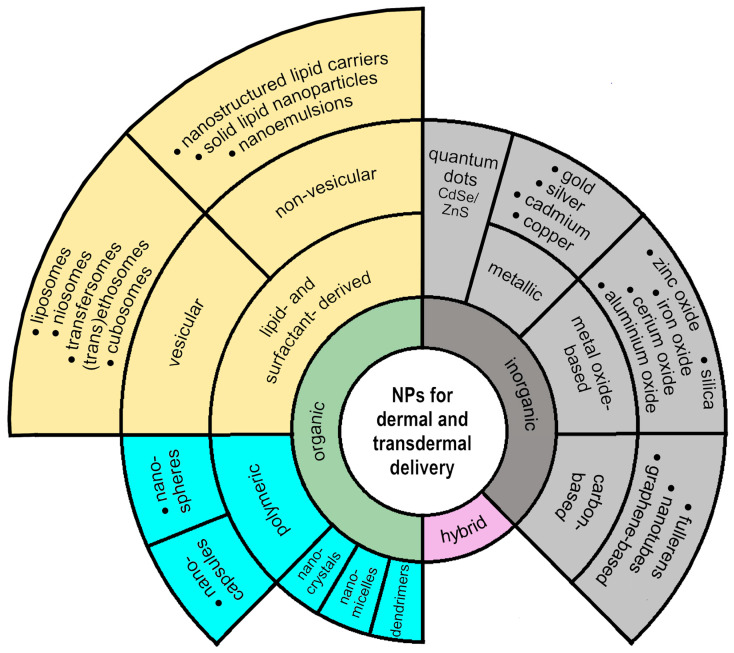
Example of nanoparticle (NP)-based carriers used as topical, dermal and transdermal drug delivery systems.

It is well known that the key to an effective dermal and transdermal drug delivery system lies in the correct choice of formulation type, as the degree of drug diffusion through the skin is influenced by its composition and structural characteristics. These formulations usually include different types of gels (hydrogels, oleogels, bigels, emulgels, nanogels), emulsions (microemulsions (MEs), nanoemulsions (NEs), multiple emulsions) and systems based on liquid crystals, with the addition of nanotechnologies for the incorporation of APIs into carriers such as liposomes, niosomes, cerosomes, nanostructured lipid carriers (NLCs), solid lipid nanoparticles (SLNs), polymer nanoparticles, micelles, dendrimers or carbon nanotubes [49]. Examples of advanced nanoparticulate delivery systems used to improve skin penetration and retention and dermal and transdermal drug delivery are presented in Figure 2.

The increasing interest in the domain of novel nanoparticulate carriers as topical, dermal and transdermal delivery systems can be proven by the number of published papers in recent years in JCR-indexed journals in the Web of Science (WoS) and PubMed databases (more than 2000 results from 2020 to 2024; search query: [(nanoparticle* OR nanoparticulate) AND (dermal OR transdermal) AND (drug OR cosmetic) AND (delivery)]; query topic: title/abstract/keywords) [50,51]. In order to give a systematic overview of the recent advances and trends in the field, only peer review, open access, full text original research articles written in English were selected (531 candidate papers) before being manually filtered and analyzed (removal of duplicates, inspection of the title, abstract, or full content of the paper, application of established quality criteria) to confirm that they are in scope. A brief survey of some representative reported studies investigating nanoparticles for dermal and transdermal drug delivery, covering the period of the last five years, is presented in Table 2. This list is not exhaustive but aims to summarize the authors’ view of the latest research achievements, future directions and perspectives on this topic.

Some studies conducted by our research group [114,115,116] were related to the formulation of MEs, NEs (high- or low-energy) or in the form of SLNs or NLCs as carriers for APIs with different mechanisms of action, as follows: the NSAIDs (aceclofenac and ibuprofen), tacrolimus, sertaconazole-nitrate and curcumin with the use of chemical penetration enhancers (different monoterpenes) or physical procedures to improve penetration into the skin (solid or soluble microneedles or iontophoresis). For instance, aceclofenac MEs based on biocompatible sucrose esters (laurate or myristate) showed superiority compared to those stabilized with polysorbate 80 with different cosurfactants (isopropyl alcohol vs. Transcutol P), as demonstrated by the total amount of aceclofenac that permeated the SC and the plasma concentrations of a model drug in rats in an in vivo PK study [114]. Furthermore, when investigating the influence of the combined application of biocompatible lecithin-based NEs stabilized by sucrose esters and solid microneedles on the delivery of the same API using in vitro release/in vivo permeation tests (tape stripping and differential tape stripping on the pig ear skin), the superiority of sucrose ester-based over polysorbate-based NEs was confirmed. In addition, the ratio of plasma concentrations of aceclofenac and its major metabolite diclofenac was significantly altered in rats after administration of NEs compared to the intravenous solution of aceclofenac, which was further significantly improved after pretreatment of rat skin with microneedles, with the result that the combination of microneedles with sucrose palmitate-co-stabilized lecithin-based NEs was useful in achieving higher concentrations of the model drug in the skin, while the combination of microneedles with polysorbate 80-costabilized NEs was more successful in improving the delivery of aceclofenac into the systemic circulation [115].

Further analysis of recently published results indicates an interesting study evaluating the formulation of a transdermal therapeutic system (TTS) with ketorolac tromethamine based on jute-derived nanocrystalline cellulose used to develop a transdermal bionanocomposite patch that also contained methylcellulose and chitosan and was prepared by a solvent evaporation technique. However, this study lacks in vivo PK data for a more realistic assessment of the carrier’s success in the transdermal delivery of the model drug [117].

A review of the FDA’s clinical trials database (ClinicalTrials.Gov) [118] shows that 621 clinical trials have been approved in recent years that include the term “transdermal therapeutic system” as one of the key words, so these trials are at various stages of implementation. The amount of studies with the status “completed” or “completed with results” is 437, while 50 studies have the status “terminated” or “terminated with results” and some are marked as “withdrawn”, “suspended” or “unknown status” [118]. Most of the studies are still ongoing, and it is expected that some of them will contribute with positive results to a final approval of the tested TTS/TDDS in the near future. Despite a relatively large number of clinical trials, there are currently 34 approved TTS products on the market worldwide [119]. Searching the Orange Book (“Approved Drug Products with Therapeutic Equivalence Evaluations”) database [120], 30 innovator and 66 generic transdermal drug products were identified, of which 40 drug products were authorized for market by the Food and Drug Administration (FDA) in the last five years (6 innovator drugs, 34 generic drugs). Details of FDA-approved, mainly originator (brand name) transdermal drug products are given in Table 3.

It should be emphasized that none of the approved transdermal products are based on nanoparticulate carriers [118], although the number of original research studies and review articles addressing this topic is continuously increasing [49,119,122]. To further demonstrate the pertinent achievements in topical and transdermal drug delivery, the data from the FDA’s clinical trials database ClinicalTrials.gov [118] were manually retrieved and analyzed based on detailed inspection of the trial overview (name, summary, description) and application of specified criteria designating the quality of trial and its relevance to the review topic. An overview of selected clinical studies associated with nanoparticulate systems is provided in Table 4 that allows for identifying the current state, future trends and open issues on this topic from the authors’ point of view.

A review of available patents from 2009 [123] shows that TTS/TDDS innovations at that time were mainly based on techniques to overcome the skin barrier (physical penetration enhancers), such as microporation, needleless injectors, iontophoresis, reverse iontophoresis, phonophoresis, reverse phonophoresis, transdermal delivery based on the use of microneedles, and finally those based on micro/nanoparticle systems such as transferosomes (US20006165500), ethosomes (US5540934), US20060182799A1 and US20060182794A1), microemulsions (US5688761) and the combination of an LP-based TTS with low frequency ultrasound for improved transdermal delivery of high molecular weight proteins (insulin, erythropoietin, interferon (US5814599)). On the other hand, a search in the WIPO patent database [124] with the key terms “transdermal delivery nano” or “dermal delivery nano” reveals 130 published patents in the period 2015–2024 which consist of micro-nanostructures/carriers (MEs/NEs/NPs) and are intended for (trans)dermal delivery of the active ingredient. Some of these patents relate to the improved technology of microneedles characterized by different shapes (hemisphere, rectangle, trapezoid), different spatial orientation, variable size and higher density, i.e., with a much larger number of microneedles per unit area. However, if the phrase “transdermal drug delivery nano” is used for a search, the number is reduced to 70 patent-protected innovations or to 11 patents with the search phrase “dermal drug delivery nano” [124], again without distinguishing between local, regional and systemic drug delivery, in contrast to the approach of Ph. Eur. 11.0 [34], which makes a clear distinction between the following two product categories in the joint monograph patches: (1) cutaneous patches and (2) transdermal patches. This can be illustrated by two typical examples from the above WIPO search.

The first is WIPO 116036004 (2023), which describes a system based on a nanoparticle-loaded soluble microneedle, wherein the microneedles are composed of a matrix material of a high molecular weight polymer and, in the matrix, are nanoparticles based on modified surface poly(lactic acid-glycolic acid) copolymers with paroxetine for the treatment of CNS disorders. Microneedles were adapted to guide the transdermal delivery of nanoparticles and subsequent passage of paroxetine across the blood–brain barrier (BBB), which was confirmed in vivo (fluorescence imaging technology) [124]. The second patent (1394/DEL/2011, granted 2018) relates to the formulation of submicron lipid particles incorporated into a gel as a carrier for a combination of drugs for the effective treatment of psoriasis, where it is important to overcome the thickened barrier in the epidermis, regardless of the type of API. Although previous patents have indicated the transdermal application of antipsoriatics in the form of LP-dispersions, NEs or lipid NPs supported by improved biopharmaceutical performance, the above patent deals with the incorporation of a combination of APIs at a total concentration of 0.05% (vitamin D analogs and various corticosteroids) into submicron lipid particles derived from selected lipids (stearic acid, tristearin, trimyristine, Precirol^®^ 5 ATO, Compritol 888 ATO, cetyl palmitate, glyceryl monostearate and/or glyceryl monooleate), finally stabilizing the dispersion of the lipid particles with a Pluronic^®^ F-68 solution followed by incorporation into a hydrophilic polyacrylate-based gel [124]. Table 5 presents a brief summary of selected patents associated with nanoparticulate carriers for dermal and transdermal delivery screened out from the PATENTSCOPE database/WIPO portal over the past five years.

The above examples of recently reported original research articles, clinical studies and patent innovations retrieved from the WoS, PubMed/Medline, ClinicalTrials.gov and PATENTSCOPE databases clearly evidence continuous interest and increasing demand for nanoparticle-based (trans)dermal delivery systems, also showing that more technologies need to be combined for effective transdermal delivery, such as microneedle-based patches with nanomaterials incorporated into the product to control drug diffusion or improve drug solubility, while a physical enhancement effect is useful to further influence the systemic delivery of the drug. However, significant efforts need to be made to overcome various technological, biological and clinical challenges in developing nanoparticulate drugs, their market entrance and wide acceptance. In the following section, a summary of methods used for studying skin penetration/permeation and (trans)dermal (nano)formulations is given.

## 5. Methods for Studying Skin Penetration and Formulations

Transdermal drug delivery, as a non-invasive method of administering drugs through the skin into the systemic circulation, has many advantages over conventional routes of administration, such as avoiding first-pass metabolism, reducing side effects, improving patient compliance, and allowing controlled and sustained release of drugs [125]. However, this delivery route also faces many challenges, such as the low permeability of the skin barrier, the variability of skin properties among individuals and body sites and the potential irritation and sensitization of the skin by the drug or the formulation [23]. To overcome these challenges and optimize the design and performance of TTS/TDDS, including nanoparticulate ones, various in vivo and in vitro models have been developed to simulate the skin and evaluate the permeation and absorption of drugs across the skin.

### 5.1. In Vitro/Ex Vivo Methods

These models are commonly referred to as diffusion chambers, which consist of a donor compartment that contains the drug formulation, a receiver compartment that collects the permeated drug and a membrane that separates the two compartments and mimics the skin barrier [126]. The most widely used natural membrane is the excised human or animal skin, which provides the most realistic representation of the skin structure and function. However, the use of skin samples also poses some limitations, such as ethical issues, availability, variability, storage and handling [127].

To address these limitations, alternative membranes have been proposed, such as synthetic membranes, artificial skin models, and microfluidic devices mounted with cell cultures. These membranes aim to provide a simpler, cheaper, more reproducible and more ethical way of studying transdermal drug delivery while maintaining a reasonable correlation with the skin. In this review, we will compare and contrast three types of diffusion chambers that use the following different membranes: Franz diffusion cells, parallel artificial membrane permeability assay (PAMPA) and skin-on-a-chip (SoC) devices.

#### 5.1.1. Traditional Diffusion Chambers

Franz diffusion cells are the most widely used diffusion chambers for transdermal drug delivery research [19]. They consist of a glass or plastic cell with a cylindrical opening at the bottom where the membrane is mounted and clamped. The donor compartment is filled with the drug formulation and covered with a lid, while the receiver compartment is filled with a buffer solution and stirred by a magnetic bar or helix. The temperature of the system is maintained by a water jacket or a heating plate at 32 °C. The drug concentration in the receiver compartment is measured at different time intervals by sampling or using an online detector.

Franz diffusion cells can accommodate various types of membranes, such as human or animal skin, synthetic membranes (e.g., cellulose acetate, polydimethylsiloxane, silicone rubber, Strat-M, etc.) and artificial skin models (e.g., SkinEthic, EpiDerm) [128]. The choice of the membrane depends on the purpose and scope of the study, as well as the availability and cost of the materials. The main advantage of Franz diffusion cells is that they provide a simple and versatile platform for studying the permeation and absorption of drugs across different membranes, and they can be easily adapted to different experimental conditions and formulations [129]. Moreover, Franz diffusion cells have been extensively validated and standardized, and they are widely accepted by regulatory agencies and industry.

However, Franz diffusion cells also have some drawbacks, such as the high volume of the donor and receiver compartments, which may affect the sink conditions and the thermodynamic activity of the drug, the influence of the acceptor buffer and membrane types, and also that the donor phase is not heated [130]. Furthermore, the classical Franz diffusion cells are static and do not account for the dynamic and complex nature of the skin, including elements such as blood flow, metabolism, inflammation and wound healing. Additionally, Franz diffusion cells are labor-intensive and time-consuming, as they require manual sampling and analysis, and they have a low throughput, as they can only test one drug or formulation at a time. Table 6 shows the three main types of Franz diffusion cells and their advantages and limitations.

#### 5.1.2. Parallel Artificial Membrane Permeability Assay (PAMPA)

PAMPA is a high-throughput screening method that uses a parallel plate system to measure the passive permeability of drugs across an artificial membrane. The system for skin permeability prediction was described by Sinkó et al. [134]. The membrane is composed of a thin layer of lipid solution (e.g., phosphatidylcholine, lecithin, octanol) that is spread on a porous support (e.g., filter paper, cellulose, polycarbonate) [135]. The donor plate contains multiple wells that are filled with the drug solution and covered with the membrane, while the receiver plate contains multiple wells that are filled with a buffer solution and aligned with the donor plate. The drug concentration in the receiver plate is measured by a UV–V is spectrophotometer or a fluorescence detector [136].

PAMPA has several advantages over Franz diffusion cells and SoC devices, such as the simplicity and low cost of the membrane preparation and the system setup, the high-throughput and speed of the assay and the low consumption of the drug and additives [130]. Moreover, PAMPA can be easily modified to mimic different skin conditions and formulations, such as pH, temperature, hydration, and enhancers. Furthermore, PAMPA has been shown to have a good correlation with the permeability of drugs across human and animal skin. However, PAMPA also has some limitations, such as the inability to account for the active transport and metabolism of drugs in the skin, the variability and instability of the membrane properties and the lack of compatibility with some drug classes and formulations, such as ionic, hydrophilic and insoluble drugs [137]. Additionally, PAMPA is a crude and empirical model that does not reflect the realistic structure and function of the skin, and it may not be suitable for predicting the in vivo performance and safety of a TDDS. In conclusion, diffusion chambers are useful tools for studying the release, permeation and absorption of drugs across the skin and optimizing the design and performance of TDDS. However, each type of diffusion chamber has its own advantages and limitations, and no single model can fully capture the complexity and diversity of the skin. Therefore, it is important to select the appropriate diffusion chamber for the specific purpose and scope of the study and to compare and validate the results with other models and methods.

#### 5.1.3. Skin-on-Chip (SoC) Microfluidic Systems

SoC devices are microfluidic systems that mimic the structure and function of the skin in a miniaturized and integrated format. They consist of a microchannel network that contains a porous membrane, where a layer of skin cells (e.g., keratinocytes, fibroblasts, melanocytes, endothelial cells) is cultured [138] or skin tissue [139] is placed and exposed to the drug formulation in the upper channel while a buffer solution flows in the lower channel. The drug concentration in the lower channel can be measured by an online detector or a sampling port. The temperature, pH, oxygen, and nutrient levels of the system can be controlled by a microfluidic pump and a biosensor. To create a physiologically relevant SoC model, it is necessary to consider the main layers (epidermis and dermis) of the skin and its vasculature; selecting an appropriate biocompatible scaffold material and sufficient cell types are also crucial. The blood, lymphatic and extracellular matrix movements and shear stress should also be considered to reproduce the in vivo-like, dynamic physiological microenvironment [140]. Permeability of skins from different species or humans were compared in SoC [141] and changes in the barrier function under diseased conditions were also studied [142]. Finally, the integration of sensors in the SoC should also be considered for real-time monitoring of skin function [143].

SoC devices offer several advantages over Franz diffusion cells, such as the low volume of the donor and receiver compartments, which reduces the amount of drug and buffer required and improves the sink conditions and the thermodynamic activity of the drug. Moreover, SoC devices are dynamic and can simulate the physiological and pathological conditions of the skin, such as blood flow, metabolism, inflammation, and wound healing. Furthermore, SoC devices are automated and high-throughput, as they can test multiple drugs or formulations simultaneously and continuously. However, SoC devices also have some challenges, such as the complexity and cost of the fabrication and operation of the microfluidic system, the difficulty of maintaining the viability and functionality of the skin cells, and the lack of standardization and validation of the device design and performance. Additionally, SoC devices are still far from replicating the full complexity and diversity of the skin, such as the hair follicles, sweat glands, nerve endings and immune cells.

### 5.2. In Vivo Methods

#### 5.2.1. Transdermal Microdialysis

A versatile technique to investigate transdermal drug transport in preclinical animal models and also in humans is microdialysis, which is widely used for different purposes, like testing the effectivity of penetration enhancer methods like iontophoresis, for assessment of dermal drug formulation for the degree of penetration of active ingredients, for testing the drug-transporter interactions in the cutaneous barrier or for monitoring pathological conditions and their biomarkers in the skin [144]. During the last few decades, the application field of this method shifted from animals to humans. This is obviously a more relevant approach and does not require the scarification of experimental animals. However, these days, the early screening of topical formulations is conducted preferentially on in vitro platforms.

#### 5.2.2. Dermal Open-Flow Microperfusion

A further electronically driven in vivo method is dermal open-flow microperfusion, where minimally invasive probes placed in 0.8 µm skin depth of human thighs to enable sampling and determination of the permeated drug concentration in the dermis [145]. It is highly advantageous that transdermal delivery can be observed and evaluated in vivo with a membrane free system which has no cut off value in regard to limitation of the size of diffused molecules. However, sampling of up to 24 h is described, which can be a disadvantage for the probands, next to a certain risk of dermal infections due to the probes. This technology is undergoing the registration procedure to become a medical device in the EU.

#### 5.2.3. Tape Stripping

With this technique, SC layers are removed one after the other with adhesive tape. The skin-stripping approach is based on the concept that time profiles of the drug concentration in the SC could characterize drug absorption (uptake) and drug elimination out of the SC into the deeper skin layers, where clearance by the circulatory system occurs. Tape stripping is a commonly used technique that is minimally invasive. It is able to yield the concentration profiles of topically applied compounds in vivo by the quantification analysis of the API extracted from the tape strips [146].

### 5.3. Advanced Analytical Techniques

#### 5.3.1. ATR-FTIR Spectroscopy for Skin Penetration Studies

Apart from using confocal Raman spectroscopy (CRS), attenuated total reflection Fourier transform infrared spectroscopy (ATR-FTIR) can be used to investigate changes in skin physiology on a molecular level [147] and to monitor drug penetration into the SC [148]. For penetration studies, it is limited to the skin surface due to the limited penetration depth of the IR beam (1–2 µm), but it can be combined with tape stripping to investigate the entire SC [149]. It can be considered complementary to CRS to investigate penetration of nonvolatile molecules possessing characteristic vibrational modes and suitably distinct IR absorbances [150,151].

#### 5.3.2. AFM-IR Spectroscopy for Skin Penetration Studies

In conventional optical techniques, including ATR-FTIR and CRS, spatial resolution is limited by diffraction. Combining IR spectroscopy with atomic force microscopy (AFM), this limitation may be overcome. In AFM-IR, a pulsed laser is focused onto the sample in the proximity of the AFM-tip. When the wavelength of the IR-beam matches the absorption bands of the substance under investigation, thermal heating occurs. This leads to an expansion of the sample which, in turn, generates oscillations of the AFM-cantilever. Therefore, the chemical nature of the substance and the location can be assessed at a resolution similar to that of AFM [152]. The amplitude of the cantilever ringdown decay curve directly correlates with the IR absorbance of the sample. By scanning through the whole wavelength region of interest and measuring the peak-to-peak amplitude of the ringdown signal, an AFM-IR spectrum is generated [153].

Kemel et al. employed the method to investigate the penetration of Janus nanoparticles into the skin [154]. They describe the challenge of the method with respect to its application to skin penetration lying in sample preparation. To obtain suitable samples, the skin had to be cut into thin slices with a cryo-microtome in order to conserve its structure while obtaining thin enough slices for AFM-IR. As the sample thickness impacts the signal intensity, heterogeneous results were obtained across the samples due to their varying thickness (despite cryo-sectioning). Still, the authors were able to deduce from their analysis that Janus carriers most likely do not penetrate intact but rather dissolve in the SC lipids. AFM-IR thus seems to offer great potential in research of nanoparticle uptake into the skin once the sample preparation issues have been solved.

#### 5.3.3. Confocal Raman Spectroscopy (CRS)

Among other spectroscopic methods, CRS has been increasingly used over the past 20 years to investigate skin penetration of active ingredients, humectants and other formulation excipients. Measurement of SC thickness, as well as water and lipid profiles, is also possible [155]. A comprehensive description of the method, its benefits and challenges, as well as more technical information, can be found at Bielfeldt et al. [156]. CRS may be used in vivo as well as ex vivo or in vitro.

Why are both scattered “elastically”? When light interacts with matter, most of the photons are scattered elastically, meaning that they exhibit the same wavelength (Rayleigh scattering). A very small proportion of the photons are scattered elastically, meaning that the wavelength of the scattered light is shifted towards longer or shorter chain lengths compared to the incident light. This is called Stokes and anti-Stokes scattering, and the former is used to generate the Raman spectrum. This spectrum may be divided into two areas, the fingerprint region between 0 and approx. 2000 cm^−1^ and the high wavenumbers region between approx. 3000 and 4000 cm^−1^. Each peak is derived from the interaction of light with a specific bond within the molecule, and thus every substance gives its own specific spectrum from which it can be identified and/or tracked within a sample. Mixed samples give mixed spectra ($\vec{S}$) from which the relative contribution of a number (N) of single species ($a_{k}$) can be calculated, taking into account their individual Raman spectra (${\vec{B}}_{k}$) [157]:

(2) $$\vec{S}=\sum_{k=1}^{N}a_{k}{\vec{B}}_{k}$$

**Figure 3:**
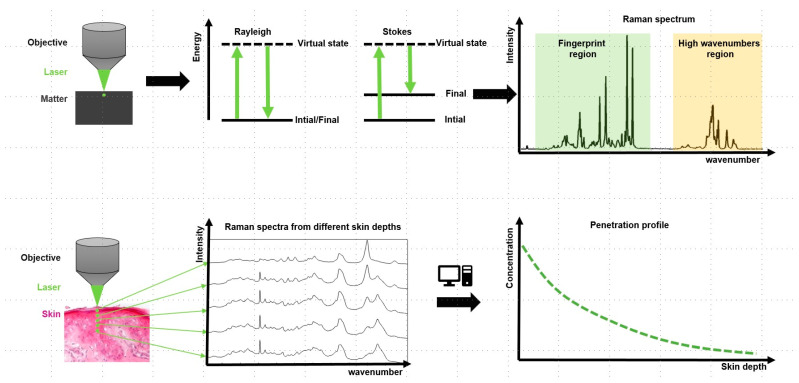
**Top row**: Laser light interacts with matter, Stokes scattering provides Raman spectrum, divided into fingerprint and high wavenumber region; **Bottom row**: laser light focused into different depths of the skin generates one spectrum per skin depth from which the penetration profile is calculated.

To monitor skin penetration, a confocal setup is used that enables focusing the light into different depths of the skin and collecting spectra only from this position by rejecting light from out of focus regions. Then, spectra are taken from consecutive positions, moving from the skin surface to deeper layers. Therefore, a number of mixed spectra are recorded that contain spectral information derived from the skin constituents as well as from the penetrated substance. Using the above formula the relative contribution of the penetrated substance can be calculated and, using a proper calibration, actual quantitative information can be obtained [158]. The principle of CRS technology is presented in Figure 3.

Until now, CRS has been used to analyze the penetration of a multitude of substances, starting with water [159], moving on to cosmetic ingredients like urea [160], retinol [158] or caffeine [161] and finally arriving at APIs like procaine [162], diclofenac [163], and vismodegib [164].

Although regulatory bodies have not yet adopted the method in their guidelines, research is generating more and more evidence to correlate CRS data to date from conventional methods like tape stripping. For example, Kourbaj et al. [161] and Krombholz et al. [162] showed that the penetration of caffeine and retinol can be monitored by CRS as well as by tape stripping and provide similar results. They described two important advantages of CRS. First, the confocality, due to which the position from which the spectra and thus the quantitative information is derived, can be adjusted precisely as opposed to tape stripping, where different amounts of SC are removed with each tape and thus the depth from which the quantitative information is derived cannot be controlled. Second, the possibility to measure the penetration kinetics of an active without the need to use excessive amounts of skin samples, like in tape stripping, where one skin sample (or more, depending on the number of repetitions) needs to be used per time point. Krombholz et al. showed that, using a modified diffusion cell that can be mounted under the objective of the Raman microscope, the penetration of actives can be monitored in real time, which gave valuable insights not only on the kinetics of the penetration of the active but also on the kinetics of penetration enhancement [165]. While these measurements were taken ex vivo and in the influx phase, Caspers et al. monitored the kinetics of actives penetration after a short incubation during the depletion phase [158]. From both types of measurements, kinetic parameters like the flux may be calculated, but they may not give the same values as the kinetics of invasion and the depletion may be different, e.g., due to the different penetration kinetics of penetration enhancers, other formulation excipients or water (as an effect of occlusion). Kourbaj et al. provide an illustrative example on how the obtained flux values differ between data collected in different phases. According to their findings, especially for fast penetrating substances, care must be taken to use suitable time points for flux calculation, as time points too close to steady state conditions will give erroneously low flux values [161]. From the current research, it can be deduced that there is an ongoing interest in the method due to the advantages detailed above. In order to enable regulatory acceptance of CRS for bioavailability and bioequivalence testing, even more evidence needs to be provided, especially when it comes to the exact methodology, including selection of time points. Furthermore, the applicability of CRS for skin penetration analysis remains to be shown for more substances from more formulations.

#### 5.3.4. Soft X-ray Scanning

X-ray scanning has been used for investigation of cells and tissues and has most recently also been utilized in skin penetration analysis. In scanning X-ray microscopy, X-rays are focused onto a small focal volume and the sample is raster-scanned to create an image. X-ray scanning has the advantage that X-rays penetrating deeper into tissues, as they exhibit shorter wavelengths than light [166]. As a result, higher spatial resolution is provided and the 2D as well as 3D analysis of complex samples, like skin, is rendered with a resolution as low as 10 nm [167]. Furthermore, X-ray scanning is a label-free technique and does not rely on staining or the use of contrast agents; thus, the penetration characteristics of a drug molecule under investigation are not altered. While high-energy (hard) X-rays create atomic fluorescence, which may be used to quantify elemental compositions and crystal lattice structures, lower-energy (soft) X-rays may be used to study magnetic states, the orientation of chemical bonds and complex chemistries such as drugs and tissue components [168]. Soft X-rays also yield improved contrasts in organic matter due to the high absorption cross sections of C, O and N shells and thus are better suited to investigating tissues. Operation of soft X-ray microscopes takes place in the “water-window”, a spectral region between the K shell absorption edges of C (284 eV, l = 4.4 nm) and O (543 eV, l = 2.3 nm) where X-rays are absorbed significantly more strongly by organic molecules than by water; therefore, contrast is enhanced by an order of magnitude [167].

Soft X-ray scanning is almost exclusively applied to skin penetration research by one group [169,170,171,172] and instruments do not seem to be as yet readily available. Soft X-ray scanning has been used to investigate the skin penetration of dexamethasone with and without prior tape stripping and with the use of core-multishell nanocarriers [169,170]. Also, the impact of serine protease or petrolatum on the skin penetration of rapamycin has been investigated [171,172].

## 6. Physiologically Based Pharmacokinetic Modelling of the Skin Penetration

### 6.1. Homogenized Membrane Model

Physiologically based pharmacokinetic (PBPK) modeling of dermal absorption provides an alternative method for the fast and cost-effective bioavailability and bioequivalence assessment of topical drugs and cosmetic care products. One particular advantage of PBPK modeling is its robustness and flexibility. It can be easily adopted to simulate either in vitro or ex vivo permeation tests [173] or in vivo exposure studies. PBPK modelling of dermal absorption has been around for several decades and has received increasing interest in recent times [174]. Most PBPK modeling uses mechanistic equations to describe the underlying pharmacokinetics of transdermal permeation using the diffusion-partition theory, with the skin barrier being considered as a homogenized membrane. The mathematical equation describing skin solute permeation through the homogenized skin membrane is described by Fick’s law of diffusion as:

(3) $$J=-D\frac{\partial c}{\partial y}$$

where *J* is the flux and *c* and *D* are concentration and diffusivity, respectively. Early PBPK modeling of skin penetration was limited to the steady-state of in vitro permeation tests. The flux of skin penetration $J_{ss}$ has been calculated by the following equation:

(4) $$J_{ss}=\frac{P_{sc/w}D_{sc}}{h_{sc}}c_{v}$$

Here $D_{sc}$, $P_{sc/w}$ and $h_{sc}$ are the homogenised diffusion coefficient, partition coefficient and thickness of the SC, respectively, and *c_v_* is the concentration of permeant in the donor vehicle.

The maximum flux of skin penetration $J_{ss-max}$ is reached when the concentration *c_v_* in the vehicle reaches saturated solubility S:

(5) $$J_{ss-max}=\frac{P_{sc/w}D_{sc}}{h_{sc}}S$$

PBPK modeling of skin permeation across the homogenized SC barrier in transient conditions has been reported using either numerical solutions or analytical solutions [175]. Under the infinite dose condition, where the accumulative amount of tested solute permeated into the skin is less than 10% during a 24 h permeation test, the Crank equation of diffusion across a planar membrane can be adapted [176]:

(6) $$c_{sc}\left(y\right)=\frac{P_{SC/w}}{P_{v/w}}c_{v}\left[1-\frac{y}{h_{sc}}+\frac{\pi }{2}\sum_{1}^{\infty }\frac{\cos\left(n\pi \right)}{n}\sin\left(\frac{n\pi y}{h_{sc}}\right)\exp\left(-D_{sc}n^{2}\pi^{2}\frac{t}{h_{sc}^{2}}\right)\right]$$

from which the accumulative amount of permeant diffused across the skin barrier is obtained as:

(7) $$Q_{t}\left(y\right)=\frac{P_{SC/w}}{P_{v/w}}c_{d}\left[\frac{D_{sc}}{h_{sc}}t-\frac{h_{sc}}{6}-\frac{2h_{sc}}{\pi^{2}}\sum_{1}^{\infty }\frac{\cos\left(n\pi \right)}{n}\exp\left(-D_{sc}n^{2}\pi^{2}\frac{t}{h_{sc}^{2}}\right)\right]$$

Here $P_{v/w}$ is the vehicle-water partition coefficient of permeant. Under an infinite dose condition, typically with a vehicle thickness of more than 150 µm [177], the accumulative amount approaches a steady state after a lag time, and the relationship is described as:

(8) $$Q_{t}\left(y\right)=\frac{K_{Sc/w}}{K_{v/w}}c_{d}\left(\frac{D_{sc}}{h_{sc}}t-\frac{h_{sc}}{6}\right)$$

where the lag time is given as:

(9) $$t_{lag}=\frac{h_{sc}^{2}}{6D_{sc}}$$

In recent years, there has been increasing interest in permeation testing under finite dose conditions to mimic more closely the in-use conditions, typically with a vehicle thickness of less than 50 µm, e.g., [178]. Analytical solutions for skin absorption and permeation under finite dose conditions have been also reported, and the readers are referred to elsewhere, for example, the study reported by Anissimov and Roberts using the Laplace transformation [175].

### 6.2. Microscopic Modeling of SC Permeation Pathways

At the microscopic level, solute transfer through the SC barrier is 3D. To model the 3D microscopic effect, the governing equation for solute transfer can be formulated separately for the SC lipid, corneocytes, viable dermis, dermis and appendage domains. For the “brick and mortar” microstructure of the SC, an analytical expression for the permeability of the tortuous lipid pathway was presented by Michaels et al. [179] as follows:

(10) $$P_{l}=\frac{K_{l/w}D_{l-lat}}{h_{sc}\tau }=\frac{K_{l/w}D_{l-lat}}{h_{sc}}\;\frac{t+g}{\left(\frac{{K_{l/w}D}_{l-lat}}{D_{c}}t+g\right)}$$

(11) $$\tau =\frac{d}{2g}\left[1+\frac{d+g}{2\left(t+g\right)}\right]$$

Johnson et al. evaluated the diffusion path length of the SC intercellular lipid for a 3D geometry of the “bricks and mortar” and proposed a different equation for the tortuosity factor, as follows [180]:

(12) $$\tau =1+\frac{2g}{h_{sc}}ln\left(\frac{d}{2s}\right)+\frac{Ndt}{h_{sc}s}+{\left(\frac{d}{1+\omega }\right)}^{2}\left(\frac{\omega }{h_{sc}g}\right)\left(N-1\right)$$

where N is the number of the corneocyte layers, *ω = d_m_*/*d_n_* is the offset ratio and t≈g is the intercellular gap in horizontal and vertical directions.

For solute diffusion in SC corneocytes, binding of the solute to keratin needs to be considered. Reported studies suggest that solute binding to keratin correlates to the hydrophobicity [181]. Corneocytes have been often modeled as porous media consisting of primarily fibrous keratin [182]. The volume fraction of the porous fluid depends on the hydration level. Most microscopic PBPK modeling of percutaneous absorption requires a numerical solution. Each of the heterogenous domains of the skin barrier are meshed to sufficiently fine elements or grids. The following generalized numerical scheme can be adopted to calculate the solute diffusion from each meshed grid i to its neighboring grid j by the following numerical scheme:

(13) $$q_{ij}=\frac{A_{ij}}{\frac{\delta_{i}}{D_{i}}+\frac{K_{i/w}\delta_{j}}{{K_{j/w}D}_{j}}}\left(c_{i}-\frac{K_{i/w}}{K_{j/w}}c_{j}\right)$$

where $q_{ij}$ is the flux of solute from grid i to grid j, $K_{i/w}$ and $K_{j/w}$ are the partition coefficients of solute between grid i and water and between grid j and water, $c_{i}$ and $c_{j}\;$ are the concentrations of solute, $\delta_{i}$ and $\delta_{j}$ are the diffusion lengths of grid i and j,  and $D_{i}$ and $D_{j}$ are the diffusivity of solute in grid i and j respectively. For each grid, Equation (11) is substituted into the following mass balance equation:

(14) $$\frac{{dc}_{i}}{dt}=-\frac{\sum_{j}q_{ij}}{V_{i}}$$

**Figure 4:**
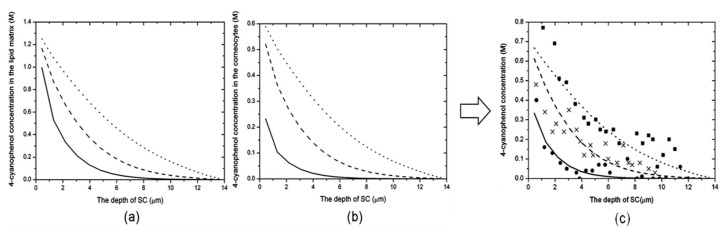
Microscopic PBPK modeling of 4-cyanophenol disposition in SC lipids (**a**) and corneocytes (**b**) domains under in vivo exposure to healthy volunteers at 1 min(-------) (•), 5 min (- - -) (**×**) and 15 min (………) (■). Predicted overall disposition (**c**) by combining both lipids and corneocytes domains showed good agreement with tape striping data. Figure modified from [183] with permission of John Wiley and sons at the License Number 5810360623095.

The above equations are solved using numerical methods. Figure 4 shows an example of microscopic PBPK modeling of a 4-cyanophenol disposition in the SC lipids and corneocytes where experimental data were obtained by tape striping on health volunteers [183]. There is a significant amount of 4-cyanophenol permeated into SC corneocytes. The overall amount predicted by the microscopic PBPK modeling agreed well with the experimental data.

**Figure 5:**
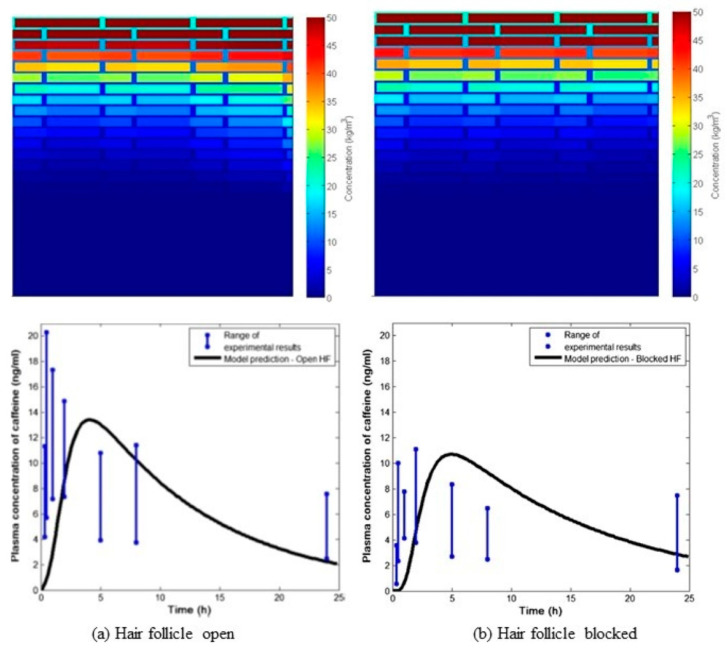
Microscopic PBPK modeling of caffeine deposition in SC lipids and corneocytes domains after 20 min application to the chest of health volunteers (above). The predicted effect of hair follicle open (**a**) and blocked (**b**) on systemic bioavailability agreed well with experimental data. Figure adopted from [163] open access.

Figure 5 shows another example of the microscopic PBPK modeling of caffeine deposition in SC lipids and corneocyte domains, as well as the systemic bioavailability, where caffeine was applied on the chests of healthy volunteers [184]. Here, the effect of hair follicle pore blocking was investigated. An important aspect revealed by the microscopic PBPK modeling is that caffeine diffused faster in the hair follicle gap than in the SC lipids. The hair follicle pathway not only contributed to the ca. 25% systemic bioavailability of the caffeine but also led to faster delivery with an earlier arrival of $c_{max}$.

Other microscopic PBPK modeling studies of transdermal permeation have also been reported elsewhere [185]. The so-called two-phase model of Wang et al. [185] is very similar to the microscopic PBPK model reported by Chen et al. [182,183]. The two-phase model of Wang et al. has been primarily limited to fitting to skin permeability data [185]. Despite PBPK modeling having been reported on from time to time for several decades, and despite significant progress being made, challenges still remain. Most PBPK modeling is limited to case-by-case scenarios with the input parameters being fitted to experimental data. Extrapolation to other exposure scenarios is difficult. Another challenge is the lack of understanding of formulation impacts. PBPK modeling of how complex formulation ingredients and product microstructures modulate percutaneous absorption is rather limited. Solute transport in the cornified envelope of the lipid domain is heterogenous, anisotropic and dependent on the lipid bilayer structure. Molecular dynamic simulations of solute permeation through SC lipid bilayer have been reported recently [186]. Of particular interest is how hydration and exposure to the chemicals of skin care and dermatological products, as well as environmental pollutants, affect the integrity of SC lipids. It has been revealed that, while the integrity of SC lipids is retained when exposed to different hydration and glycerol, fatty acid extraction is observed when SC lipids are exposed to ethanol [187]. It can be envisaged that advancements in molecular dynamics and multiscale methods will make PBPK modeling of dermal absorption a more widely adopted tool.

## 7. Outlook—Current Approaches and Research Trends

Classic strategies to dermally administer drugs with small molecular masses and high potencies will continue to rely on passive diffusion and/or formulation strategies using chemical enhancers. However, delivery of biotech drugs, i.e., large hydrophilic peptides and macromolecules, will in many cases rely on the use of more advanced enhancers and/or active enhancement strategies, especially for delivery of genetic material such as DNA, RNA and/or vaccines [125]. The examples below highlight the potential of TDDSs to complement or replace existing pharmaceutic delivery systems for administration of large molecular mass cargoes, providing benefits such as high specificity, reduced side-effects, and good patient compliance.

Shin et al. [188] evaluated a hydrophobic cell-penetrating peptide (CPP, MTD 1067) for delivery of protein cargoes of various sizes, including growth-hormone-releasing-hexapeptide-6, a truncated form of insulin-like growth factor-I, and a platelet-derived growth factor. MTD-conjugated cargoes were non-toxic and exhibited biological activities that were identical or improved when compared to the unconjugated control. Confocal microscopy showed higher levels in the dermis (4.4-, 18.8-, and 32.9-times higher than the control), emphasizing the potential of CPP-conjugation for transdermal delivery of macromolecular drugs. In another recent approach, Kang et al. [189] developed functionalized nanocarriers with CPPs for enhancement of transdermal delivery. The basic vehicles exhibited specific self-assembly and penetration efficiency in dependence of their amino acid composition, indicating that tailor-made vehicles can be designed for delivery of different actives.

Rentzsch et al. [28] explored the use of conventional intradermal injection and NanoPass microneedles to deliver a small-molecule specific ligand of Langerin conjugated to a model protein antigen into human skin explants. Selective loading of Langerhans cells in the epidermis was achieved with both methods. However, conventional intradermal injections, albeit approved by regulatory agencies and cost-efficient, require trained personnel and bear the risk of needle-caused injury and low patient compliance. The FDA-approved NanoPass microneedles allow for safe and painless administration without prolonged training and thus provide relevant advantages as intradermal delivery systems for vaccination. Van der Straeten et al. [190] reported successful intradermal delivery of a mRNA vaccine in a lipid nanoparticle (LNP) vehicle using a thermostable microneedle patch; a long-term immune response comparable to intramuscular administration was observed in mice. This study highlights the potential of TDDSs to facilitate vaccination programs also in countries with insufficient infrastructures in regard to handling thermo-sensitive administration systems.

The goal of needle-free transdermal application of biomacromolecules was likewise targeted by Zhu et al. [191], who developed a transdermal delivery platform based on biocompatible fluorocarbon modified chitosan, proposed for antibody and antigen delivery. Mouse models were used to test the penetration potential and induced immune responses, suggesting promising results.

## 8. Conclusions

Topical, dermal and transdermal delivery presents an emerging route of drug administration, owing to its inherent advantages/benefits, such as non-invasive application, by-pass of rapid first-pass metabolism, avoidance/reduction in off-site toxicity and systemic adverse effects, increasing bioavailability, possibility of controlled and sustained drug release, and, hence, improved therapeutic efficacy and patient compliance. Looking at the pool of reported studies, clinical trials and patent innovations in recent years, it is apparent that many works and efforts have been devoted to the development and application of advanced topical/transdermal formulations and methods, as well as to skin penetration-enhancing techniques. Although many advancements have been already achieved in the field, formulation and optimization of safe, effective and marketable nanoparticle-based topical and transdermal delivery systems still remains challenging, keeping in mind elements such as skin barrier properties, low skin permeability, interindividual and inter-site variability, potential for skin irritation and sensitization, among others. This further imposes the need for employment of innovative nanotechnological strategies and advanced analytical techniques to support the development of nano-drug transdermal formulations through comprehensive evaluation and prediction of their skin penetration/permeation and, particularly, in vivo fate.

As a future direction, beside the above-mentioned innovations, the administration of large molecular mass cargoes with novel drug delivery systems, e.g., the intradermal delivery of mRNA vaccines, is expected to provide benefits such as high specificity, reduced side effects and good patient compliance.

## Figures and Tables

**Table 1:** Overview of liquid and semisolid topical formulations classified by the Ph. Eur. 11.0 [34].

	Solution	Emulsion	Suspension	Gel	Ointment	Cream	Paste
No of phases, *n*	*n* = 1	*n* > 1	*n = 2*	*n* = 1	*n* = 1	*n* > 1	*n* > 1
Consistency	liquid	liquid	liquid	semisolid	semisolid	semisolid	semisolid
Main excipients	water; alcohol or oil	water; alcohol; oil; emulsifier	water; alcohol or oil; finely dispersed solids	water; alcohol; gel former	oil; white soft paraffin; wax; constancy agents; emulsifier (in water absorbing ointments)	water; oil; white soft paraffin; wax; constancy agents; emulsifier	ointment or cream
Subgroups and phasing	hydrophilic; lipophilic	hydrophilic (oil-in-water, O/W); lipophilic (water-in-oil, W/O)	hydrophilic; lipophilic	hydrophilic (hydrogel); lipophilic (oleogel)	hydrophilic; lipophilic; water absorbing: lipophilic	hydrophilic; lipophilic	hydrophilic; lipophilic
Galenic characteristics	high spreadability, especially on hair-rich skin	high spreadability, especially on hair-rich skin	high spreadability, especially on hair-rich skin	hydrogels: cooling effect; oleogels: occlusive	hydrophilic: cooling effect; water absorbing: partially occlusive, water uptake	hydrophilic: “lighter” skin feeling; lipophilic: partially occlusive	more solid than corresponding ointment or cream

**Table 2:** Summary of selected recent research studies exploring nanoparticle-based topical, dermal and transdermal drug delivery systems (publication years 2020–2024).

Delivery System	Description/ Excipients	Active Ingredient (Category)	Route	Dosage Form	Purpose	Ref.
Lipid- and surfactant-based vesicular NPs	Liposomes/ phospholipids, cholesterol	Tetramethylpyrazine (anti-inflammatory, antioxidant)	Transdermal	Gel	Development of multifunctional hydrogel drug delivery system based on tetramethylpyrazine-loaded liposomes surrounded by sodium alginate-chitosan hydrogel for treating atopic dermatitis	[52]
	Liposomes/ anionic: DPPC, DSPE-mPEG2000, cholesterol; cationic: DOTAP, DOPE	Dexamethasone (corticosteroid)	Transdermal	Microneedle patch	Design and evaluation of dexamethasone-loaded cationic and anionic liposomes integrated in hyaluronic acid-based microneedles, to enhance intracellular drug delivery efficiency in psoriatic skin, by regulating size and surface charge of drug-loaded liposomes	[53]
	Liposomes/ cholesterol, hydrogenated soybean phosphatidylcholine, DSPE-mPEG2000	Doxorubicin hydrochloride (chemotherapeutic); Indocyanine green (photosensitizer)	Transdermal	Microneedle patch	Design and characterization of a near-infrared (NIR) light-activatable dissolving, polyvinylpyrrolidone microneedle system incorporating liposomes co-loaded with the active oxygen species (ROS)-responsive doxorubicin prodrug and the photosensitizer indocyanine green, to allow a transdermal delivery method with controllable drug release/activation and effective imaging guidance for melanoma ablation	[54]
	Aspasomes– ascorbyl palmitate-based liposomes/ ascorbyl palmitate, cholesterol, phospholipid	Itraconazole (antifungal agent)	Topical	Cream	Design and clinical evaluation of itraconazole-loaded aspasomes (newer antioxidant generation of liposomes) enclosed in topical oil-in-water cream as a potent nano-sized delivery system for effective treatment of dermal fungal infections	[55]
	Niosomes/ cholesterol, Span^®^ 60	Apigenin (herbal bioactive compound; antimicrobial, antioxidant, anti-cancer, anti-inflammatory effects)	Transdermal	Gel	Development of apigenin-loaded niosomes incorporated into chitosan gel to improve transdermal delivery and therapeutic efficacy	[56]
	Niosomes/ cholesterol, Span^®^ 60, Span^®^ 80	Amphotericin B (antifungal); Pentamidine (antiparasitic)	Transdermal	Gel	Preparation of amphotericin B-pentamidine-loaded niosomes and incorporation into chitosan gel for better skin penetration, sustained release and improved anti-leishmanial activity in the treatment of cutaneous leishmaniasis	[57]
	Niosomes/ cholesterol, Span^®^ 60	Thymol (herbal antimicrobial agent)	Topical	Gel	Development and evaluation of thymol-encapsulated niosomes incorporated into gelatin methacryloyl polymeric hydrogel to improve thymol antibacterial and anti-biofilm activity and wound healing	[58]
	Spanlastics—elastic niosomes/ Span^®^ 60; Tween^®^ 20, 60 or 80, or Brij^®^ 35, 58, or 97	Miconazole nitrate (antifungal)	Topical	Gel	Design and evaluation of miconazole nitrate-loaded spanlastics incorporated in carbopol gel for topical application in deeply sated skin fungal infections	[59]
	Transethosomes/ Phospholipon^®^ 90G, Tween^®^ 80, ethanol	Metformin hydrochloride (antidiabetic)	Transdermal	Gel	Design, optimization and evaluation of metformin hydrochloride transethosomes incorporated into chitosan gel to provide sustained release, reduce side effects and improve transdermal drug delivery and therapeutic effect	[60]
	Transethosomes/ lecithin, oleic acid, ethanol	Miconazole nitrate (antifungal)	Topical	Gel	Development and evaluation of miconazole nitrate-loaded transethosomes incorporated into carbopol gel to enhance skin permeability and antifungal activity	[61]
	Transfersomes/ Phospholipon^®^ 90G, Tween^®^ 80	Nitazoxanide (antiparasitic); Quercetin (natural flavonoid; antioxidant, anti-inflammatory, anti-cancer, anti-leishmanial effects)	Topical	Gel	Development, characterization and evaluation of nitazoxanide and quercetin co-loaded nanotransfersomes incorporated into chitosan gel for the treatment of cutaneous leishmaniasis, with the aim to achieve passive targeting of dermal macrophages and improve therapeutic effect	[62]
	CPP-modified transfersomes/ soybean phospholipid, DOTAP, sodium cholate hydrate; modification: CPP Stearyl-R5H3)	Lycorine (isoquinoline alkaloid; anti-cancer antiviral, antibacterial, and anti-inflammatory effects)	Topical	Gel	Development and evaluation of lycorine-oleic acid ionic complex-loaded CPP-modified cationic transfersomes incorporated into carbopol gel, for topical treatment of cutaneous squamous cell carcinoma, aiming to enhance skin and tumor permeability and drug delivery	[63]
	Invasomes/ soy lecithin, terpene (citronella) oil, ethanol	Luliconazole (antifungal)	Topical	Gel	Development and characterization of luliconazole-loaded soft invasomes incorporated into carbopol gel to increase drug skin penetration and topical antifungal efficacy	[64]
	Cubosomes/ glyceryl monooleate, Pluronic^®^ F-127	Doxorubicin hydrochloride (chemotherapeutic); Indocyanine green (photothermal agent)	Transdermal	Gel	Development of doxorubicin and indocyanine green co-loaded cubosomes (incorporatd in xanthan gum gel), as a promising transdermal nanocarrier to enhance drug permeation across the skin and improve efficacy of melanoma treatment by combination chemo-photothermal therapy	[65]
	Cubosomes/ glyceryl monooleate, Pluronic^®^ F-127	β-Sitosterol (natural phytosterol; for promoting hair growth)	Transdermal	Microneedle patch	Design and evaluation of β-sitosterol loaded cubosomes integrated with dissolving microneedles (hyaluronic acid and polyvinyl pirolidone K90 matrix) as a novel dermal delivery system for β-sitosterol, to obtain increased skin penetration and controlled release for the treatment of alopecia	[66]
	Cubosomes/ glyceryl monooleate, polyvinyl alcohol 6000	Febuxostat (xanthine oxidase inhibitor, for hyperuricemia treatment)	Transdermal	Microneedle patch	Design and evaluation of febuxostat-loaded cubosomes entrapped into fast dissolving microneedles to enhance drug permeation, bioavailability and effectiveness, and avoid side effect in the treatment of gout	[67]
	PEGylated cerosomes—ceramide-enclosed tubular vesicles/ egg yolk phosphatidylcholine, stearylamine, ceramide IIIB; PEGylated surfactant: Brij^®^ 52 or Brij^®^ 97	Fenticonazole nitrate (antifungal agent)	Topical	Suspension (vesicles dispersion)	Design and evaluation of fenticonazole nitrate-loaded PEGylated cerosomes as a novel topical drug delivery system for effective treatment of fungal skin infections	[68]
Lipid- and surfactant-based non-vesicular NPs	SLNs/ glyceryl monostearate, Tween^®^ 80	Paroxetine (antidepressant)	Transdermal	Patch	Fabrication of paroxetine-loaded SLNs incorporated in transdermal patch to increase drug absorption and bioavailability	[69]
	SLNs/ Phospholipon^®^ 90 H, glyceryl monostearate, Tween^®^ 20	Sulconazole (antifungal)	Topical	Gel	Formulation design, in vitro and in vivo evaluation of sulconazole-loaded SLNs incorporated into carbopol-based topical gel for enhanced skin penetration and antifungal activity	[70]
	SLNs/ Compritol^®^ 888 ATO, poloxamer 188	Nicotine (for nicotine replacement therapy)	Transdermal	Gel	Development and evaluation of nicotine–stearic acid conjugate-loaded SLNs incorporated in hydroxypropyl methylcellulose (HPMC) gel for nicotine transdermal delivery in the treatment of smoking cessation	[71]
	SLNs/ Precirol^®^ ATO 5, poloxamer 188;	Hexylresorcinol (whitening agent); Ginger oil (inhibition of tyrosinase activity and microftalmia transcription factor, antioxidant, anti-inflammatory agent)	Topical	Suspension (NPs dispersion)	Development, in vitro and in vivo evaluation of stable lipid NPs as carriers for co-delivery of 4-hexylresorcinol and ginger oil for the effective cutaneous hyperpigmentation treatment	[72]
	NLCs/ Precirol^®^ ATO 5, Labrafac™ lipophile WL 1349 or ginger oil, poloxamer 188, sucrose distearate or Span^®^ 85					
	NLCs/ glyceryl behenate, PEG-8 caprylic/capric glycerides, Tween^®^ 20, poloxamer 188, or phosphatidylcholine, chitosan	Thymol (antimicrobial natural compound)	Topical	Gel	Development, optimization and evaluation of thymol-loaded functionalized/surface-modified NLCs dispersed in carbomer gelling systems for topical use against acne vulgaris	[73]
	NLCs/ Softisan^®^ 649 and Miglyol^®^ 812-based, freeze-dried	Genistein (natural isoflavone; antioxidant)	Topical	Gel	Development and characterization of gel-like matrix containing genistein-loaded NLCs to improve drug retention on the skin; evaluation of the developed genistein delivery system for potential use in skin protection and UV radiation-associated skin diseases	[74]
	NLCs/ Precirol^®^ ATO 5; geranium oil, tee tree oil, lavender oil; Tween^®^ 80	Luteolin (antipsoriatic)	Topical	Suspension (NPs dispersion)	Development and evaluation of novel luteolin-loaded NLCs comprising different anti-inflammatory oils to enhance luteolin skin deposition and augment its functionality in the treatment of psoriasis	[75]
	NLCs/ Precirol^®^ ATO 5, oleic acid, Tween^®^ 80	Ranolazine (anti-anginal, anti-ischemic agent)	Transdermal	Gel	Development and evaluation of ranolazine-loaded NLCs incorporated into transdermal carbopol gel, to enhance drug bioavailability and improve the treatment efficacy of angina pectoris	[76]
	NLCs/ Precirol^®^ ATO 5; castor oil/PEG-8 caprylic/capric glycerides; Tween^®^ 80	Pranoprofen (NSAID)	Topical	Gel	Desgn and evaluation of pranoprofen-loaded NLCs incorporated into Carbopol^®^ 940- or Sepigel^®^ 305-based gels as effective topical delivery system for the treatment of local skin inflammation	[77]
	NLCs/ glyceryl monostearate; Capryol^®^ 90; poloxamer 188)	Resveratrol (natural polyphenol; anti-cancer, antioxidant, anti-inflammatory, neuroprotective, cardioprotective, and anti-aging activities)	Transdermal	Microneedle patch	Fabrication and characterization of resveratrol-loaded NLCs incorporated into carbopol gel and administered via microneedle array delivery system to enable localized drug delivery and improved efficacy for breast cancer therapy	[78]
	NLCs/ cetyl palmitate; oleic acid; Transcutol P, limonene; polysorbate 80, polysorbate 20)	Capsaicin (active alkaloid of chilli peppers, *Capsicum* extract; analgesic, to treat pain-related symptoms)	Topical	Patch	Development and evaluation of capsaicin-loaded NLCs embedded in polyacrylic acid transdermal patches to improve skin delivery of capsaicin and reduce its skin adverse effects for the treatment of skeletomuscular and neuropathic pain	[79]
	Nanoemulsion/ medium chain triglycerides, Span^®^ 80, Tween^®^ 80;	Curcumin (anti-inflammatory, antioxidant, antimicrobial, wound-healing)	Topical	Emulsion (nano);	Development and evaluation of two types of curcumin-loaded lipid nanocarriers—nanoemulsion and NLCs—to select the most appropriate curcumin delivery platform for the treatment of skin burns	[80]
	NLCs/ medium chain triglycerides, glyceryl monostearate, Span^®^ 80, Tween^®^ 80			Suspension (NPs dispersion)		
	Nanoemulsion/ pepermint and bergamot oils; Tween^®^ 80; Transcutol^®^ P or PEG 400	Glimepiride (oral hypoglycemic drug)	Transdermal	Gel	Preparation and evaluation of glimepiride-loaded nanoemulsion incorporated into different gels (carbopol-, sodium carboxymethyl cellulose-, sodium alginate-, or HPMC-based) to enhance transdermal drug delivery and its therapeutic efficacy in diabetes treatment	[81]
	Nanoemulsion/ eucalyptus oil, Span^®^ 80, Tween^®^ 80	Mupirocin (antibiotic)	Topical	Emulgel	Development, characterization and evaluation of mupirocin nanoemulgel, that is mupirocin-loaded nanoemulsion incorporated into carbopol hydrogel, characterized by high skin deposition and enhanced antibacterial effect, for targeting skin lesions	[82]
	Nanoemulsion/ Capmul^®^ PG-8, Transcutol^®^ P, Kolliphor^®^ EL, Tween^®^ 80, propylene glycol	Bromocriptine mesylate (semisynthetic ergot alkaloid; dopaminergic agonist, for Parkinson’s disease treatment)	Transdermal	Gel	Development and characterization of bromocriptine-loaded nanoemulsion incorporated into carbopol-based gel, as effective transdermal drug delivery system to enhance drug bioavailability, reduce side effects and increase patient compliance in the management of Parkinson’s disease	[83]
	Nanoemulsion/Self-nanoemulsifying drug delivery system/ oil: garlic oil; surfactant: Tween^®^ 20/Span^®^ 20 mixture; co-surfactant: propylene glycol	Acyclovir (antiviral drug); Garlic oil (antiviral effect)	Transdermal	Film	Formulation and evaluation of acyclovir and garlic oil-loaded self-nanoemulsifying drug delivery system incorporated into hydroxypropyl cellulose transdermal film as a promising therapeutic approach in relieving cold sores symptoms	[84]
	Nanoemulsion/ evening primrose oil, Span^®^ 60, Tween^®^ 80;	Ibuprofen (non-steroidal anti-inflammatory drug, NSAID)	Transdermal	Emulsion (nano);	Development and evaluation of three types of nanoparticulate transdermal delivery systems of ibuprofen—nanoemulsion, nanoemulgel, and polymeric NPs (polycaprolactone, polyvinyl alcohol, sucrose)—aiming to enhance skin permeation, leading to improved pain relief and patient compliance	[85]
	Nanoemulgel/ nanoemulsion + Carbopol^®^ Ultrez 20			Emulgel (nano);		
Polymeric NPs	Chitosan NPs	Risedronate sodium (anti-osteoporosis drug)	Transdermal	Gel	Formulation and in vitro/ex vivo evaluation of a novel transdermal gel loaded with risedronate-chitosan NPs to treat osteoporosis	[86]
	Chitosan NPs functionalized with hyaluronic acid	Curcumin (natural polyphenol; anti-inflammatory, antioxidant, antimicrobial, wound-healing, tissue regenerating, immunomodulatory, anti-cancer effects); Quercetin (natural polyphenol; antioxidant, antifibrotic, wound-healing, anti-inflammatory activity)	Topical	Suspension (NPs dispersion)	Development and evaluation of hyaluronic acid functionalized-curcumin and quercetin co-loaded polymeric NPs to improve dug skin targeting and efficacy in the treatment of burn wounds	[87]
	L-arginine-based polyamide NPs	Thymoquinone (antipsoriatic)	Transdermal	Patch	Synthesis and characterization of novel thymoquinone-loaded L-arginine-based polyamide nanocapsules incorporated into transdermal patches for potential management of psoriasis	[88]
	PLGA NPs/ poly(lactic-co-glycolic acid) (PLGA)	Lurasidone (antipsychotic)	Transdermal	Microneedle patch	Development and evaluation of transdermal delivery system for lurasidone, namely lurasidone-loaded PLGA NPs incorporated into effervescent microneedles, to increase drug bioavailability and schizophrenia patient adherence	[89]
	PLGA NPs	Tetrakis(1-methyl-4-pyridinio)porphyrin (photosenzitizer, anti-cancer)	Transdermal	Gel miconeedles	Fabrication of drug-loaded PLGA NPs and incorporation into enzyme mediated nanocomposite hyaluronic acid-tyramine hydrogel microneedles with tunable mechanical strength and controllable transdermal efficiency in melanoma	[90]
	PLGA NPs	Risperidone (atypical antipsychotic drug)	Transdermal	System (NPs-loaded organogel)	Preparation and characterization of risperidone-loaded PLGA NPs incorporated into poloxamer lecithin organogel as a novel nanocarrier system for transdermal delivery of risperidone, in order to improve drug skin permeation and reduce side effects	[91]
	PCL NPs/ polycaprolactone (PCL), Tween^®^ 80	Betamethasone phosphate (corticosteroid); Minoxidil (hair regrowth agent, for alopecia)	Topical	Suspension (NPs dispersion)	Development and comparative evaluation of batamethasone and minoxidil co-loaded polymeric and lipidic NPs for enhanced targeted follicular drug delivery in treating alopecia	[92]
	Chitosan-coated PLGA NPs	Prednisolone (corticosteroid)	Topical	Gel	Development and evaluation of prednisolone-loaded polymeric NPs incorporated into poloxamer hydrogel to improve anti-inflammatory action and reduce side effects in the treatment of chronic actinic dermatitis	[93]
	Chitosan-grafted polymeric NPs/ chitosan, poly(glycidyl methacrylate-co-N-isopropyl acrylamide—Poly(GMA-co-NIPAAm, Pluronic^®^ F-127	ISX9 (neurogenesis inducer, stimulates hair follicle growth)	Transdermal	System	Synthesis and characterization of ISX9-loaded thermoresponsive, biopolymer-based nanoparticulate drug delivery system, comprising chitosan-grafted Poly(GMA-co-NIPAAm) as the shell component and Pluronic^®^ F-127 as the core polymer, to treat androgenetic alopecia	[94]
	Chitosan-coated nanocapsules/ chitosan, Pluronic^®^ F-127	Retinyl palmitate (antioxidant, anti-inflammatory, anti-wrinkle)	Topical	Lyophilized powder for suspension	Development and in vitro evaluation of vitamin A derivative-loaded chitosan-coated nanocapsules for effective skin delivery of retinyl palmitate, with enhanced stability, skin penetration and efficacy	[95]
	Functionalised chitosan NPs/ chitosan, functional additives: hyaluronic acid sodium salt and/or collagen	Alpha arbutin (natural hydroquinone derivative; skin whitening agent, tyrosinase inhibitor)	Topical	Gel	Formulation and evaluation of alpha arbutin-loaded functionalized chitosan NPs encapsulated in carbopol hydrogel to enhance drug delivery and therapeutic efficacy in melasma treatment	[96]
	Eudragit L 100 NPs	Budesonide (corticosteroid)	Topical	Gel	Development of pH-sensitive, budesonide-loaded NPs embedded into hydrogels for local therapy of atopic dermatitis in pediatric population	[97]
	Silk fibroin NPs	Triptorelin (gonadotropin-releasing hormone agonist)	Transdermal	Microneedle patch	Development and evaluation of silk fibroin-based microneedles for delivery of triptorelin-loaded silk fibroin NPs to improve relative bioavailability and tolerability of triptorelin for women undergoing assisted reproductive technology	[98]
	Polymeric NPs/fiber system/ Poly (3-hydroxybutyrate-co-3-hydroxyvalerate fiber mashes decorated with PLGA NPs	Dexamethasone (corticosteroid, anti-inflammatory)	Transdermal/tympanic	Patch	Proof of concept of a dual drug-loaded patch based on rhodamine-loaded NPs and dexamethasone-loaded ultra-fine fibers, as an effective transdermal drug delivery system for the treatment of chronic inflammatory conditions or sudden sensorineural hearing loss	[99]
	Human serum albumin-based protein NPs	Basic fibroblast growth factor, bFGF (accelerating angiogenesis and skin tissue regeneration, by promoting proliferation and migration of dermal cells for wound healing)	Topical	Suspension (NPs dispersion)	Development of recombinant bFGF protein-loaded human serum albumin NPs as a promising delivery platform for improved bFGF stability, sustained release and enhanced wound healing and tissue regeneration	[100]
	Ferulic acid-derived lignin NPs	Minoxidil (stimulation of hair follicle regrowth, for alopecia)	Transdermal	Nanocomposite paste	Development and evaluation of carboxymethyl cellulose/hyaluronic acid/polyvinylpyrrolidone-based composite paste system incorporating minoxidil-loaded NPs and valproic acid for the treatment of androgenic alopecia, with using roller-microneedles to create mechanical microchannels and enhance transdermal drug delivery and efficiency	[101]
Micelles	Polymeric micelles/ D-α-tocopherol polyethylene glycol 1000 succinate (TPGS)	TAK-441 (hedgehog pathway inhibitor, anti-cancer)	Topical	Gel	Development and evaluation of TAK-441-loaded TPGS micelles incorporated into HPMC hydrogel to enhance cutaneous drug delivery and deposition, thereby reducing systemic adverse effects, for the treatment of basal cell carcinoma	[102]
Nanocrystals	Nanocrystals/ Nanosuspension/ drug; stabilizer: poly(vinyl alcohol), sodium dodecyl sulfate, or Tween^®^ 80	Ivermectin (antiparasitic)	Topical	Cream	Preparation and characterization of ivermectin nanocrystales to improve drug solubility, skin penetration and efficacy when applied topically in the treatment of parasitic infections	[103]
	Nanocrystals/ Nanosuspension/ drug; polysorbate 60	Azelaic acid (antibacterial, anti-acne, anti-inflammatory, keratolytic activity)	Topical	Gel	Preparation and evaluation of azelaic acid nanocrystals-loaded in situ hidrogel (Pluronic^®^ F-127, hyaluronic acid), characterized by improved efficacy and favorable safety for the treatment of acne vulgaris	[104]
Metallic NPs	Hyaluronic acid-coated zeolitic imidazolate framework-8 NPs	Fucoidan, zinc (antimicrobial agent)	Dermal (subcutaneous)	Microneedle patch	Synthesis of multifunctional fucoidan-loaded zinc-metal organic framework-encapsulated microneedles for methicillin-resistant *Staphylococcus aureus*-infected wound healing	[105]
	Gold NPs and silica NPs	Chloramphenicol (antibiotic)	Topical	Gel	Investigation of carbopol-based hydrogels loaded with chloramphenicol-coated gold and silica NPs, as a promising strategy to increase dermal drug delivery, improve antimicrobial activity and reduce the concentration of antibiotic, thus improving its safety and efficacy	[106]
Metal oxide NPs	Starch-coated iron oxide NPs	Doxorubicin hydrochloride (chemotherapeutic)	Transdermal	Patch	Fabrication of a non-invasive, innovative transdermal patch loaded with greenly synthesized iron oxide NPs conjugated to doxorubicin, to improve drug delivery for breast cancer treatment	[107]
	Bovine serum albumin (BSA)-modified cerium-manganese oxide NPs	Methotrexate (anti-rheumatic)	Transdermal	Microneedle patch	Synthesis of BSA-modified cerium-manganese oxide NPs loaded with methotrexate and integrated into soluble hyaluronic acid-based microneedles, for targeting macrophages in the treatment of rheumatoid arthritis	[108]
Hybrid NPs	Phospholipid-calcium carbonate hybrid NPs/ lipids: soy lecithin, triglyceride monostearate	Tofacitinib citrate (Janus kinase inhibitor)	Topical	Suspension (NPs dispersion)	Design of phospholipid-coated amorphous calcium carbonate NPs for targeted delivery of tofacitinib citrate to hair follicles, for prevention and treatment of chemotherapy-induced alopecia	[109]
	Gold NPs conjugated lecithin-chitosan hybrid NPs	Tacrolimus (immunosuppressive, antiproliferative, anti-inflammatory agent)	Topical	Suspension (NPs dispersion)	Conjugation of gold NPs with tacrolimus-loaded chitosan NPs or tacrolimus-loaded hybrid lecithin-chitosan NPs, as a promising drug delivery system for the treatment of psoriasis	[110]
	Lecithin/chitosan NPs	Pentoxifylline (anti-inflammatory drug)	Topical	Sponge (gel)	Development and characterization of lignocellulose nanocomposite sponge (hydrogel) incorporating pentoxifylline-loaded lecithin/chitosan NPs to control drug release for accelerated wound healing	[111]
	ZnO/Ag NPs (silver NPs dispersed in zinc oxide mesoporous microspheres), decorated with polycaprolactone-polyethylene glycol-polycaprolactone (PCL-PEG-PCL) nanomicelles	Methotrexate (immunosuppressive regulator, antipsoriatic)	Topical	Gel	Design and evaluation of multifunctional nanocomposite-based carbopol hydrogel comprising methotrexate-loaded ZnO/Ag NPs embellished with PCL-PEG-PCL nanomicelles to enhance therapeutic efficacy for psoriasis via synergistic combined therapy	[112]
	Zinc oxide (ZnO) NPs/polyacrylonitrile electrospun nanofibers	Ibuprofen (NSAID)	Transdermal	System (electrospun nanofiber membrane)	Development and evaluation of dual-stimulus (pH and temperature) responsive transdermal drug delivery system containing ibuprofen-loaded ZnO NPs and polyacrylonitrile electrospun nanofiber membranes, aiming to achieve increased and sustained drug release	[113]

NPs: nanoparticles; DPPC: 1,2-dipalmitoyl-sn-glycero-3-phosphocholine; DSPE-mPEG2000: 1,2-distearoyl-sn-glycero-3-phosphoethanolamine-N-(metoxy[polyethyleneglycol]-2000; DOTAP: 2,3-dioleoyloxy-propyl-trimethylammonium-chloride; DOPE: 1,2-dioleoyl-sn-glycero-3-phosphoethanolamine; NLCs: nanostructured lipid carriers; SLNs: solid lipid nanoparticles.

**Table 3:** Summary of FDA approved transdermal drug products [120,121].

Active Ingredient (Therapeutic Class)	Proprietary Name (Marketing Status)	Dosage Form	Indication	Product Characteristics	Applicant	Approval Year
Asenapine (atypical antipsychotic)	Secuado^®^ (Rx)	Transdermal system	Treatment of schizophrenia in adults	Description: translucent rounded square patch with a printed backing on one side and a release liner on the other; Excipients: alicyclic saturated hydrocarbon resin, butylated hydroxytoluene, isopropyl palmitate, maleate salts (monosodium maleate and disodium maleate), mineral oil, polyester film backing, polyisobutylene, silicone-treated polyester release liner, sodium acetate anhydrous, styrene-isoprene-styrene block copolymer; Duration of application: 24 h; Application site: hip, abdomen, upper arm, or upper back area	Hisamitsu Pharmaceutical Co., Inc. (Saga, Japan)	2019 (Patent expiration: 2033)
Buprenorphine (partial opioid agonist/antagonist)	Butrans^®^ (Rx)	Film, extended release	Management of moderate to severe chronic pain in patients requiring a continuous, around-the-clock opioid analgesic for an extended period of time	Description: rectangular transdermal system/patch, polymer matrix type (outer backing layer, adhesive film layer, foil layer between adhesive and drug/polymer adhesive matrix, peel-off release liner); Excipients: levulinic acid, oleyl oleate, povidone, polyacrylate cross-linked with aluminum; Duration of application: 7 days; Application site: upper outer arm, upper chest, upper back, or the side of the chest	Purdue Pharma L.P. (Stamford, CT, USA)	2010, 2013, 2014 *
Clonidine (centrally acting hypotensive agent, alpha-agonist)	Catapres-TTS^®^ (Rx)	Transdermal system	Treatment of hypertension	Description: square, tan adhesive patch—multi-layered film, reservoir-type (backing layer, drug reservoir, control membrane, adhesive layer, slit release liner); Composition, excipients: backing layer of pigmented polyester and aluminum film; drug reservoir of clonidine, mineral oil, polyisobutylene, and colloidal silicon dioxide; microporous polypropylene control membrane; adhesive layer of clonidine, mineral oil, polyisobutylene, and colloidal silicone dioxide; Duration of application: 7 days; Application site: upper outer arm or chest	Lavipharm S.A. (Peania Attica, Greece)	1984
Dextroamphetamine (central nervous system stimulant)	Xelstrym^®^ (Rx)	Transdermal system	Treatment of attention deficit hyperactivity disorder (ADHD) in adults and pediatric patients 6 years and older	Description: translucent transdermal system with a printed backing on one side and a release liner on the other, adhesive matrix type (backing layer, adhesive matrix containing drug, peel-of release liner); Excipients: acrylic adhesives, green ink, polyester/polyurethane backing, silicone-coated polyester release liner; Duration of application: up to 9 h in a day; Application site: hip, upper arm, chest, upper back, or flank	Noven Pharmaceuticals, Inc. (Miami, FL, USA)	2022 (Patent expiration: 2025, 2033, 2042)
Donepezil hydrochloride (acetylcholinesterase inhibitor)	Adlarity^®^ (Rx)	Transdermal system	Treatment of mild, moderate, and severe dementia of the Alzheimer’s type	Description: rectangular six-layer laminate with rounded corners and a tan colored backing layer, multilaminate polymer matrix system (overlay backing/adhesive layer without drug, separating layer, drug matrix, microporous membrane, contact adhesive, release liner); Excipients: acrylate copolymer, ascorbyl palmitate, crospovidone, glycerol, lauryl lactate, polypropylene membrane, sodium bicarbonate, sorbitan monolaurate, triethyl citrate; Duration of application: 7 days; Application site: upper or lower back (avoiding the spine), upper buttocks, or upper outer thigh	Corium, Inc. (Boston, MA, USA)	2022 (Patent expiration: 2037, 2038)
Estradiol (estrogen hormone)	Climara^®^ (Rx)	Film, extended release	Treatment of moderate to severe vasomotor symptoms and symptoms of vulvar and vaginal atrophy due to menopause; treatment of hypoestrogenism due to hypogonadism, castration, or primary ovarian failure; prevention of postmenopausal osteoporosis	Description: translucent transdermal system, monolithic adhesive matrix type (backing layer, drug/adhesive layer—adhesive matrix containing estradiol, protective liner); Excipients: acrylate copolymer adhesive, fatty acid esters, polyethylene backing; Duration of application: 7 days; Application site: lower abdomen (below the umbilicus) or upper quadrant of the buttock	Bayer Healthcare Pharmaceuticals, Inc. (Berlin, Germany)	1994, 1998, 1999, 2003 *
	Minivelle^®^ (Rx)	Film, extended release	Treatment of moderate to severe vasomotor symptoms due to menopause; prevention of postmenopausal osteoporosis	Description: transdermal system, multipolymeric adhesive matrix type (backing layer, adhesive layer containing drug, protective liner); Excipients: polyolefin laminate backing, acrylic adhesive, silicone adhesive, oleyl alcohol, povidone, dipropylene glycol, polyester release liner; Duration of application: 3–4 days (twice weekly); Application site: lower abdomen (below the umbilicus) or buttocks	Noven Pharmaceuticals, Inc. (Miami, FL, USA)	2012, 2014 (Patent expiration: 2028, 2030)
	Divigel^®^ (Rx)	Gel	Treatment of moderate to severe vasomotor symptoms associated with menopause	Description: clear, colorless, smooth gel, odorless when dry, hydrophilic gel; Excipients: carbomer, ethanol, propylene glycol, purified water, triethanolamine; Duration of application: once daily; Application site: upper thigh (in a thin layer, to a small area 200 cm^2^)	Vertical Pharmaceuticals, LLC (Alpharetta, GA, USA)	2007
	Elestrin™ (Rx)	Gel, metered	Treatment of moderate to severe vasomotor symptoms associated with menopause	Description: colorless, non-staining hydroalcoholic topical gel supplied in a non-aerosol, metered-dose pump container, hydrophilic gel; Excipients: ethanol, propylene glycol, diethylene glycol monoethyl ether, carbomer 940, triethanolamine, edetate disodium, purified water; Duration of application: 1 or 2 pump actuations per day; Application site: upper arm to shoulder (in a thin layer, to area about 320 cm^2^)	Mylan Specialty L.P. (Morgantown, WV, USA)	2006
	EstroGel^®^ (Rx)	Gel, metered	Treatment of moderate to severe vasomotor symptoms due to menopause; treatment of moderate to severe symptoms of vulvar and vaginal atrophy due to menopause	Description: clear, colorless gel, odorless when dry, supplied in a metered-dose pump, hydrophilic gel; Excipients: purified water, alcohol, triethanolamine, carbomer 934P; Duration of application: 1 pump depression per day; Application site: entire arm from wrist to shoulder (in a thin layer)	ASCEND Therapeutics US, LLC (Herndon, VA, USA)	2004
	Evamist (Rx)	Transdermal spray	Treatment of moderate to severe vasomotor symptoms due to menopause	Description: rapidly drying solution supplied in a metered-dose pump; Excipients: octisalate, alcohol; Duration of application: 1–3 sprays per day, each morning; Application site: inner forearm	Padagis US, LLC (Allegan, MI, USA)	2007
	Menostar™ (Rx)	Transdermal system	Prevention of postmenopausal osteoporosis	Description: oval, translucent transdermal system/patch, monolithic adhesive matrix type (backing layer, drug/adhesive layer—acrylate adhesive matrix containing estradiol, protective liner); Excipients: acrylate copolymer adhesive, fatty acid esters, polyethylene backing; Duration of application: 7 days; Application site: lower abdomen (below the belly button)	Bayer Healthcare Pharmaceuticals. Inc. (Berlin, Germany)	2004
	Vivelle-Dot^®^ (Rx)	Transdermal system	Treatment of moderate to severe vasomotor symptoms and symptoms of vulvar and vaginal atrophy due to menopause; treatment of hypoestrogenism due to hypogonadism, castration, or primary ovarian failure; prevention of postmenopausal osteoporosis	Description: transdermal system, multipolymeric adhesive matrix type (backing layer, adhesive containing drug, protective liner); Composition, excipients: translucent polyolefin backing film; adhesive formulation containing estradiol, acrylic adhesive, silicone adhesive, oleyl alcohol, povidone, and dipropylene glycol; polyester release liner; Duration of application: 3–4 days (twice weekly); Application site: lower abdomen or buttocks	Sandoz. Inc. (Basel, Switzerland)	1999, 2002
Estradiol (estrogen hormone); Levonorgestrel (progestogen hormone)	Climara Pro^®^ (Rx)	Film, extended release	Treatment of moderate to severe vasomotor symptoms due to menopause; prevention of postmenopausal osteoporosis	Description: adhesive-based matrix transdermal system/patch (backing layer, drug-in-adhesive layer, protective liner); Excipients: acrylate copolymer adhesive, polyvinylpyrrolidone/vinyl acetate copolymer, polyethylene backing; Duration of application: 7 days; Application site: lower abdomen or buttocks	Bayer Healthcare Pharmaceuticals, Inc. (Berlin, Germany)	2003
Estradiol (estrogen hormone); Norethindrone acetate (progestational agent, progestin)	CombiPatch^®^ (Rx)	Film, extended release	Treatment of moderate to severe vasomotor symptoms due to menopause; treatment of vulvar and vaginal atrophy; treatment of hypoestrogenism due to hypogonadism, castration, or primary ovarian failure	Description: adhesive-based matrix transdermal system/patch (translucent polyolefin backing layer, drugs-in-adhesive layer, protective liner); Excipients: acrylic adhesive, silicone adhesive, oleic acid, povidone, dipropylene glycol, polyester release protective liner; Duration of application: 3–4 days (twice weekly); Application site: lower abdomen	Noven Pharmaceuticals, Inc. (Miami, FL, USA)	1998
Ethinyl estradiol (estrogen); Levonorgestrel (progestin)	Twirla^®^ (Rx)	Transdermal system	Method of contraception for use in women of reproductive potential with a BMI < 30 kg/m^2^, for whom a combined hormonal contraceptive is appropriate	Description: circular beige colored transdermal system/patch, matrix type (woven peripheral backing layer etched with drugs, inactive peripheral acrylic adhesive layer, inactive peripheral polyisobutylene adhesive layer, internal separating membrane, active adhesive matrix, polyester release liner); Excipients: acrylic adhesives, capric acid, copovidone, crospovidone, dimethyl sulfoxide, ethyl lactate, lauryl lactate, polybutene, polyester internal membrane, polyester release liner, polyisobutylene adhesives, woven polyester backing membrane; Duration of application: 7 days (once weekly, for 3 consecutive weeks); Application site: abdomen, buttock, or upper torso (excluding breasts)	Agile Therapeutics, Inc. (Princeton, NJ, USA)	2020 (Patent expiration: 2028)
Ethinyl estradiol (estrogen); Norelgestromin (progestin)	Xulane™ (Rx)	Film, extended release	Prevention of pregnancy in women who elect to use a transdermal patch as a method of contraception	Description: thin, matrix-type transdermal system (backing layer, adhesive layer containing hormones, release liner); Excipients: polyisobutene adhesive, crospovidone, mineral oil, non-woven polyester fabric, oleyl alcohol, dipropylene glycol, polyester backing film laminate, polyester release liner with a fluoropolymer coating; Duration of application: 7 days (once weekly, for 3 consecutive weeks); Application site: upper outer arm, abdomen, buttock, or back	Mylan Technologies, Inc. (Saint Albans Town, VT, USA)	2014
Fentanyl (opioid analgesic)	Fentanyl-12, -25, -37, -50, -62, -75, -87, -100 (Rx)	Film, extended release	Treatment of moderate to severe chronic pain in patients requiring a continuous, around-the-clock opioid analgesic for an extended period of time	Description: translucent rectangular transdermal system/patch with rounded corners, adhesive matrix type (backing layer, adhesive layer containing drug, protective liner); Excipients: dimethicone, silicone adhesive, polyolefin film backing; Duration of application: 72 h; Application site: chest, back, flank, or upper arm	Mylan Technologies, Inc. (Saint Albans Town, VT, USA)	2005, 2007, 2014 *
Granisetron (serotonin-3 (5-HT_3_) receptor antagonist)	Sancuso^®^ (Rx)	Film, extended release	Prevention of nausea and vomiting in adults receiving moderately and/or highly emetogenic hemotherapy for up to 5 consecutive days	Description: translucent, rectangular transdermal system with rounded corners, polymer matrix type (backing polyester layer, drug matrix (acrylate-vinyl acetate copolymer), siliconized polyester release liner); Excipients: acrylate-vinyl acetate copolymer, polyester, titanium dioxide, polyamide resin, polyethylene wax; Duration of application: up to 7 days; Application site: upper outer arm	Cumberland Pharmaceuticals, Inc. (Nashville, TN, USA)	2008 (Patent expiration: 2025)
Methylphenidate (central nervous system stimulant)	Daytrana^®^ (Rx)	Film, extended release	Treatment of ADHD in pediatric patients 6 to 17 years of age	Description: adhesive-based matrix transdermal system (outside backing, multipolymeric adhesive containing drug, protective liner); Composition, excipients: polyester/ethylene vinyl acetate laminate film backing, proprietary adhesive formulation incorporating DOT Matrix™ (Noven Pharmaceuticals, Inc.) transdermal technology containing acrylic adhesive and silicone adhesive, fluoropolymer-coated polyester protective liner; Duration of application: up to 9 h; Application site: hip area	Noven Pharmaceuticals, Inc. (Miami, FL, USA)	2006 (Patent expiration: 2025)
Nitroglycerin (organic nitrate)	Nitro-Dur^®^ (Rx)	Film, extended release	Prevention of angina pectoris due to coronary artery disease	Description: transdermal infusion system, flat unit designed, adhesive matrix type (impermeable backing layer, drug/adhesive layer); Excipients: acrylic-based polymer adhesives with a resinous cross-linking agent; Duration of application: 12–14 h; Application site: reasonably hair-free area (chest, shoulder, upper arm, or back)	USpharma, Ltd. (Miami Lakes, FL, USA)	1995
Oxybutynin (anticholinergic agent, muscarinic antagonist)	Oxytrol^®^ (Rx)	Film, extended release	Treatment of overactive bladder in men with symptoms of urge urinary incontinence, urgency, and frequency	Description: matrix-type transdermal system/patch (backing film, adhesive/drug layer, overlapped release liner); Composition, excipients: polyester/ethylene-vinyl acetate backing film, acrylic adhesive, triacetin, siliconized polyester film; Duration of application: 3–4 days; Application site: abdomen, hip, or buttock	Allergan Sales LLC (Irvine, CA, USA)	2003
Rivastigmine (acetylcholinesterase inhibitor)	Exelon^®^ (Rx)	Film, extended release	Treatment of mild, moderate, and severe dementia of the Alzheimer’s type; Treatment of mild-to-moderate dementia associated with Parkinson’s disease	Description: transdermal patch, 4-layer laminate, polymer matrix type (backing layer, drug product (acrylic) matrix, adhesive (silicon) matrix, overlapping release liner); Excipients: acrylic copolymer, poly (butylmethacrylate, methylmethacrylate), silicone adhesive applied to a flexible polymer backing film, silicone oil, vitamin E; Duration of application: 24 h; Application site: upper or lower back, upper arm, or chest	Sandoz, Inc. (Basel, Switzerland)	2007, 2012 *
Rotigotine (dopamine agonist)	Neupro^®^ (Rx)	Film, extended release	Treatment of Parkinson’s disease; Treatment of moderate-to-severe primary restless legs syndrome	Description: thin, tan-colored transdermal system, adhesive matrix type (backing film, self-adhesive drug matrix layer, protective liner); Excipients: aluminized polyester film, ascorbyl palmitate, povidone, silicone adhesive, sodium metabisulfite, DL-alpha-tocopherol, fluoropolymer-coated polyester film; Duration of application: 24 h; Application site: abdomen, thigh, hip, flank, shoulder, or upper arm	UCB, Inc. (Smyrna, GA, USA)	2007, 2012 (Patent expiration: 2025, 2027, 2030, 2032)
Scopolamine (anticholinergic)	Transderm Scop^®^ (Rx)	Transdermal system	Prevention of nausea and vomiting associated with motion sickness; prevention of post-operative nausea and vomiting associated with recovery from anesthesia and/or opiate analgesia and surgery	Description: tan-colored, circle shaped transdermal system/patch, reservoir-type, multilaminate polymer matrix system (backing membrane, drug layer, rate controlling membrane, drug containing contact layer, release liner); Excipients: light mineral oil, polyisobutylene, polypropylene, aluminized polyester film; Duration of application: up to 3 days; Application site: hairless area behind one ear	Baxter Healthcare Corp. (Deerfield, IL, USA)	Approved prior to 1982
Selegiline (monoamine oxidase inhibitor)	Emsam^®^ (Rx)	Film, extended release	Treatment of major depressive disorder	Description: matrix-type transdermal system (backing film, adhesive/drug layer, release liner); Excipients: acrylic adhesive, ethylene vinyl acetate, polyethylene, polyester, polyurethane, silicone coated polyester; Duration of application: 24 h; Application site: upper torso (below neck and above waist), upper thigh, or upper arm (outer surface)	Somerset Pharmaceuticals, Inc. (Morgantown, WV, USA)	2006
Testosterone (androgen hormone)	Testim^®^ (Rx)	Gel	Testosterone replacement therapy in males for conditions associated with a deficiency or absence of endogenous testosterone: primary hypogonadism, hypogonadotropic hypogonadism	Description: clear to translucent hydroalcoholic gel for topical use; Excipients: purified water, pentadecalactone, carbopol, acrylates, propylene glycol, glycerin, polyethylene glycol, ethanol (74%), tromethamine; Duration of application: one tube once daily; Application site: shoulders and/or upper arms	Endo Operations, Ltd. (Dublin, Ireland)	2002
	Vogelxo^®^ (Rx)	Gel; gel, metered	Testosterone replacement therapy in males for conditions associated with a deficiency or absence of endogenous testosterone: primary hypogonadism, hypogonadotropic hypogonadism	Description: clear to translucent hydroalcoholic gel for topical use, available in unit-dose tube, unit-dose packet, and metered-dose pump; Excipients: carbomer copolymer type B, carbomer homopolymer type C, diisopropyl adipate, ethyl alcohol, glycerin, methyl laurate, oleyl alcohol, polyethylene glycol, propylene glycol, purified water, tromethamine; Duration of application: one tube, or one packet, or 4 pump actuations, once daily; Application site: shoulders and/or upper arms	Upsher-Smith Laboratories, LLC (Maple Grove, MN, USA)	2014 (Patent expiration: 2034)
	AndroGel^®^ (Rx)	Gel, metered	Testosterone replacement therapy in males for conditions associated with a deficiency or absence of endogenous testosterone: primary hypogonadism, hypogonadotropic hypogonadism	Description: clear, colorless, hydroalcoholic gel available in a metered-dose pump; Excipients: carbopol 980, ethyl alcohol, isopropyl myristate, purified water, sodium hydroxide; Duration of application: 2 pump actuations, once daily, in the morning; Application site: shoulders and upper arms	Besins Healthcare Ireland, Ltd. (Dublin, Ireland)	2011 (Patent expiration: 2026)
Nicotine (stop smoking aid)	Habitrol^®^ (OTC)	Film, extended release	Support in quitting smoking by reducing nicotine withdrawal symptoms, including nicotine craving	Description: transdermal system/patch, matrix type; Excipients: acrylate adhesive, aluminized polyester, cellulose paper, methacrylic acid copolymer; Duration of application: 24 h; Application site: upper body or outer part of arm	Dr. Reddy’s Laboratories SA (Hyderabad, India)	1999
	NicoDerm CQ^®^ (OTC)	Film, extended release	Support in quitting smoking by reducing nicotine withdrawal symptoms, including nicotine craving	Description: thin, clear or opaque transdermal system/patch, reservoir/membrane type; Excipients: ethylene vinyl acetate copolymer, polyisobutylene, high density polyethylene between clear polyester backings; Duration of application: 16 h or 24 h; Application site: any hairless body area (arm, leg, chest, back)	Chattem Inc., DBA Sanofi Consumer Healthcare (Chattanooga, TN, USA)	1996
Oxybutynin (anticholinergic agent, muscarinic antagonist)	Oxytrol^®^ for Women (OTC)	Film, extended release	Treatment of overactive bladder symptoms in adult women	Description, type: matrix-type transdermal system/patch, matrix type (backing film, drug/triacetin/adhesive matrix, two overlapped-tab release liner strips); Excipients: acrylic adhesive, polyester/ethylene-vinyl acetate film, siliconized polyester film, triacetin; Duration of application: 4 days; Application site: abdomen, hips, or buttocks	AbbVie (North Chicago, IL, USA)	2013

Marketing status: Rx—prescription, OTC—over-the-counter. * Different approval dates refer to different strengths of drug product. All listed transdermal drug products are innovator drug products, except Xulane™ (ethinyl estradiol; norelgestromin) and Fentanyl (fentanyl) that are generics.

**Table 4:** Presentation of some representative clinical studies involving nanoparticles for topical/dermal and transdermal drug and cosmetic delivery [118].

Study Title	NCT Number	Status	Condition/Disease	Intervention/Treatment (Drug, Delivery System, Device)	Phase
Topical silver nanoparticles for microbial activity	NCT03752424	Unknown	Foot infection, fungal; infection, bacterial	Silver nanoparticles loaded into topical cream	1
Capsaicin nanoparticle in patient with painful diabetic neuropathy	NCT01125215	Unknown	Painful diabetic neuropathy	Capsaicin nanoparticle cream	2 and 3
Topical fluorescent nanoparticles conjugated somatostatin analog for suppression and bioimaging breast cancer	NCT04138342	Unknown	Breast cancer; skin cancer; skin diseases	Quantum dots coated with veldoreotide: Carboxylic acid-functionalized CdS/ZnS core–shell-type quantum dots (bioimaging) conjugated to veldoreotide (somatostatin analog, anti-cancer activity), loaded in topical cream	1
Periocular rejuvenation by topical hyaluronic acid nano particles	NCT05742399	Recruiting	Tear trough eyelid deformity; dark eyelids; wrinkle	Hyaluronic acid NanoGel: drug-free hyaluronic acid nanoparticles loaded into topical gel	NA
Study of topical SOR007 ointment for cutaneous metastases	NCT03101358	Completed with results	Cutaneous metastasis	SOR007 (uncoated nanoparticle paclitaxel) ointment	1 and 2
Role of minoxidil in alopecia areata transepidermal drug delivery of minoxidil via either fractional carbon dioxide laser or microneedling versus its topical nanoparticles preparation for treatment of alopecia areata	NCT05587257	Not yet recruiting	Alopecia areata	Niosome minoxidil: minoxidil nanoparticles for topical treatment; Comparison of minoxidil transdermal delivery (devices: fractional CO2 lasser and Derma pen) and minoxidil topical spray	NA
Topical Sm29 in combination with meglumine antimoniate in the treatment of cutaneous leishmaniasis	NCT06000514	Completed	Cutaneous leishmaniasis	Sm29 protein, Schistosoma Mansoni: topical Sm29 protein in gold nanoparticles	1 and 2
Trial to assess the potency of SOR007 ointment in a psoriasis plaque test	NCT03004339	Completed	Plaque psoriasis	SOR007 (uncoated nanoparticle paclitaxel) ointment	1
Clinical assessment of oxiconazole nitrate solid lipid nanoparticles loaded gel	NCT03823040	Completed	Tinea	Oxiconazole nitrate-loaded solid lipid nanoparticles incorporated into carbopol gel	1
A new approach in laser surgery using the regenerative solution in children diagnosed with vascular pathology (DOUBLE-SKIN)	NCT04999618	Completed	Vascular diseases; vascular malformation; capillary malformation-arteriovenous malformation; port-wine stain; Sturge-Weber syndrome; vascular tumor	Haemoblock: Regenerative solution comprising silver nanoparticles (bactericidal and bacteriostatic effects), albumin coat and polyacrylate matrix, delivered with transdermal patch	4
Enhanced epidermal antigen specific immunotherapy trial-1 (EE-ASI-1)	NCT02837094	Completed	Type 1 diabetes	Gold particle-peptide injection: C19-A3 GNP (peptide fragment related to insulin) attached to gold nanoparticles, administered intradermally via Nanopass microneedles	1
A sunscreen based on bioadhesive nanoparticles	NCT02668536	Completed	Melanoma; UV ray skin damage	Bioadhesive nanoparticle sunscreen: UV filters encapsulated in skin-adhesive nanoparticles as delivery vehicle	1
Pilot study—Putative penetration of nanoparticles in sunscreen in intact or sunburned skin	NCT01552135	Completed	Healthy skin; sunburned skin	Sunscreen lotion containing titanium dioxide nanoparticles	NA
Effects of magnetic tape on the autonomic nervous system	NCT05504369	Completed	Lower back pain	Magnetic tape: magnetic nanoparticles in tape	NA
Evaluation of diabetic foot wound healing using hydrogel/nano silver-based dressing vs. traditional dressing	NCT04834245	Completed	Diabetes mellitus	Hydrogel/nano silver-based dressing	NA
Efficacy of topical liposomal form of drugs in cutaneous leishmaniasis	NCT01050777	Completed	Cutaneous leishmaniasis	Liposomal formulation of paromomycin sulfate; Liposomal formulation of meglumine antimonate (Glucantime); Liposomal meglumine antimonate	Early phase 1
Study of SOR007 ointment for actinic keratosis	NCT03083470	Completed with results	Actinic keratosis	SOR007 (uncoated nanoparticulate paclitaxel) ointment	2
Retinyl palmitate-loaded ethosomes in acne vulgaris	NCT04080869	Completed	Facial acne vulgaris	Retinyl palmitate-loaded topical ethosomes	2
Formulation and clinical evaluation of ethosomal and liposomal preparations of anthralin in psoriasis	NCT03348462	Completed	Psoriasis vulgaris	Anthralin-loaded liposomes; Anthralin-loaded ethosomes (short contact topical application)	4
Efficacy of Manuka honey nanoformulation in the treatment of acne vulgaris	NCT06175819	Not yet recruiting	Acne vulgaris	Manuka honey (MGO 850, UMF + 20)-loaded topical nanoformulation, with high content of non-peroxidase methylglyoxal (major antibacterial component of Manuka honey)	NA
Superficial basal cell cancer’s photodynamic therapy: comparing three photosensitizers: HAL and BF-200 ALA versus MAL	NCT02367547	Active, not recruiting	Neoplasms, basal cell; carcinoma, basal cell; photochemotherapy	Aminolevulinic acid nanoemulsion (BF-200 ALA) compared to hexylaminolevulinate (HAL) cream and methylaminolevulinate (MAL) cream	1 and 2
Clinical assessment of voriconazole self nano emulsifying drug delivery system intermediate gel	NCT04110860	Completed	Tinea versicolor	Voriconazole self-nanoemulsifying drug delivery system intermediate gel: voriconazole nanoemulsion for topical application	2
Clinical assessment of itraconazole self nano emulsifying drug delivery system intermediate gel	NCT04110834	Completed	Tinea versicolor	Itraconazole self-nanoemulsifying drug delivery system intermediate gel: itraconazole nanoemulsion for topical application	2
Proof-of concept study of topical 3%-diclofenac-nanoemulsion cream for knee OA pain	NCT00484120	Completed	Osteoarthritis of the knee	Diclofenac nanoemulsion topical cream	2
Nanoparticulate versus micronized steroids delivery for transdermal hormone replacement therapy (Nanoparticle)	NCT02467673	Completed	Menopausal syndrome	Nanoparticulate 17β-estradiol + progesterone	2
Transdermal HRT in relieving postmenopausal symptoms (THRT)	NCT02033512	Completed	Menopause	Transdermal nanofomulation: nanostructured formulation of estriol + estradiol, transdermal gel	2
Transdermal testosterone nanoemulsion in women libido (Biolipid/B2)	NCT02445716	Unknown	Menopause	Transdermal nanoemulsion of testosterone (compared to placebo nanoemulsion)	2

The terms used for the database search included the following: “nanoparticles for skin drug delivery” (45 results), “nanoparticles for dermal drug delivery” (four results), “nanoparticles for transdermal drug delivery” (four results), “nanoparticle topical” (17 results), “nanoparticle skin” (eight results), “nanoparticle dermal” (five results), “nanoparticle cutaneous” (five results), “nanoparticle transdermal” (three results), “skin nano” (66 results), “topical nano” (22 results), “dermal nano” (seven results), “transdermal nano” (one result) (accession on 08.05.2024); NA: not applicable.

**Table 5:** Overview of some recent international and national patents dealing with nanoparticle-based systems for dermal and transdermal drug delivery [124].

Title	Publication Number	Brief Summary/Description	Applicant(s)/ Inventor(s)	Publication Year
Topical compositions and methods of preparing the same	US20240082118	Formulation and preparation of dermatological (pharmaceutical or cosmetic) compositions containing nanoelements with water-insoluble thermoplastic collagen-synthesis stimulating compound/polymer, dispersed as solid nanoparticles or liquid nanodroplets in a polar carrier, to target sites within the skin or allow transdermal delivery of such compounds	Landa Labs (2012) Ltd. (Rehovot, Israel)/Sagi Abramovich, Gal Avidor, Benzion Landa	2024
Photothermal microneedle patch containing gold nanoparticles	WO/2024/014624	Development of skin-soluble, photothermal microneedle patch comprising drug or cosmetic formulation formed on a film comprising gold nanoparticles, with maximally efficient delivery of lipophilic substance into the skin	Kumoh National Institute of Technology Industry-Academic Cooperation Foundation/Eue Soon Jang	2024
Methotrexate and Ce6 combined transdermal drug delivery system as well as preparation method and application thereof	CN117379395	Development and characterization of aminated, hollow mesoporous silicon nanoparticles loaded with methotrexate and Ce6 and coated with hyaluronic acid, to obtain combined transdermal drug delivery system for effective and safe treatment of psoriasis	Huashan Hospital, Fudan University/Wang Zhicheng, Lu Min, Zhao Gang, Fan Zhijia	2024
Hyaluronate nanofabric sheet and manufacturing method thereof	WO/2023/191385	Formulation and preparation (electrospinning) of hyaluronate nanofabric sheet with excellent percutaneous absorption and physical properties, to be used as mask patches for topical uses/delivery systems	Jinwoo Bio Co., Ltd. (Yongin-si, Republic of Korea)/Dong Keon Kweon, Myoung Han Lee, Jong Soo Kim, Joo Yeon Hong	2023
Microneedle patch loaded with multimode driving compound as well as preparation method and application of microneedle patch	CN116763714	Development of microneedle patch loaded with multimode driving compound comprising a magnetic nanocore and mesoporous shell layer, loaded with anti-inflammatory drug, inflammation-responsive aerogenesis compound (prepared from L-arginine), and coated with photo-thermal responsive compound (polydopamine) for effective transdermal delivery into inflammatory tissue	The Affiliated People’s Hospital of Ningbo University, Ningbo Institute of Materials Technology and Engineering, Chinese Academy of Sciences/Wu Manxiang. Li Qiang, Chen Tianxiang, Wu Aiguo, Xie Dong, Wang Lianfu	2023
Biodegradable microneedle patch for transdermal gene delivery	US20230256219	Development of plasmid DNA-encapsulated poly(beta-amino ester) nanoparticles embedded in gelatin methacryloyl-based microneedle patch for the successful local and controlled/sustained transdermal delivery of plasmid DNA for tissue regeneration, cancer therapy, and other applications	The Regents of the University of California/Alireza Khademhosseini, Wujin Sun	2023
Long-acting gel preparation containing arsenic trioxide nano-liposome for treating psoriasis and preparation method of long-acting gel preparation	CN116270425	Formulation and preparation of long-acting gel loaded with arsenic trioxide liposomes as effective delivery system for the treatment of psoriasis, showing improved drug stability, sustained and controlled drug release and enhanced therapeutic efficacy	Harbin Medical University/Hai Xin, Zhao Yilei, Liu Liang, Ji Fengqi	2023
Preparation method, product and application of transdermal self-assembly nano-drug	CN116211788	Preparation of transdermal self-assembly nano-drug co-loaded with 6-mercaptopurine and programmed death receptor-1 (6-MP/Zn/PD-1), through self-assembled position reaction between 6-mercaptopurine, zinc acetate and programmed death receptor-1, and surface-modified with arginine-rich dendrimer; obtained nano-drug could be incorporated into gel for effective transdermal delivery and synergistic effect of chemotherapy and immunization	Zhejiang University Hangzhou International Science and Technology Innovation Center/Qin Yating, Wang Xi, Li Yaping, Wang Yan	2023
Deformable nano-scale vehicles (DNV) for transblood–brain barrier, transmucosal and transdermal drug delivery	JP2023075276	Preparation of deformable nano-scale drug delivery vehicles (micelles, liposomes) comprising amphipathic vesicle-forming lipids (phospholipids, cholesterol) and non-ionic surfactants (Span 80, Tween 20, Brij^®^ 76, 78, 96 or 721), possibly functionalized with a polymer (polyethylene glycol, cellulose, modified cellulose), useful for the transdermal delivery of various therapeutic agents (flavonoid, isoflavonoid, neoflavonoid, resveratrol or resveratrol analog, quinone oxido-reductase inhibitor, bisphosphonate, antibody, aptamer or miRNA)	Regents of the University of California/Varghese John, Nishimura Ichiro, Naren Subbiah, Jesus Campagna, Patricia R Spilman, Mohammad Parvez Alam	2023
Macrophage membrane bionic light immune nano drug delivery system and preparation method thereof	CN116172977	Preparation of innovative nano drug delivery system comprising chemotherapeutic drug and photoresponsive agent co-loaded cationic liposomes and macrophage membrane bionic nanoparticles incorporated into soluble microneedles, by combining nanotechnology and transdermal drug delivery technology, for the treatment of breast cancer solid tumors	China Pharmaceutical University/Wang Wei, Yin Yue, Tang Lu	2023
Tofacitinib nano microneedle preparation as well as preparation method and application thereof	CN116115552	Preparation and application of tofacitinib-loaded soluble microneedles as promising nano drug delivery system that enables effective drug skin penetration, improved therapeutic efficacy, while reducing side effects	Weifang Medical University/Zhang Weifen, Zhao Yanyan, Guo Xindong, Zhang Jingjing, Ma Weiyuan, Qu Yan	2023
Device for delivery of rheumatoid arthritis medication	US20230137871	Fabrication of microneedles array device, including nanostructures on a surface of microneedles, for delivery of a rheumatoid arthritis drug across a dermal barrier	Sorrento Therapeutics, Inc. (San Diego, CA, USA)/Russell Frederick Ross	2023
Soluble microneedle carrying nanoparticles as well as preparation method and application of soluble microneedles	CN116036004	Preparation and application of nanoparticle-loaded soluble microneedles containing polymeric matrix and nanopreparation (paroxetine-loaded poly[lactic acid-glycolic acid] nanoparticles) as carrier for transdermal drug delivery and treatment of central nervous system diseases	Guangdong Pharmaceutical University, Machuang Future [Guangzhou] Medicine Technology Co., Ltd. (Guangzhou, China)/Ban Junfeng, Li Baohua, Yuan Pinghui, Li Feihong, Liao Liqi	2023
Antibacterial nano transdermal delivery system and preparation method thereof	CN116019785	Development and preparation of macrophage membrane modified liposomes loaded with phototherapy agent and vancomycin and further incorporated into microneedle transdermal delivery system to improve/control drug release and accumulation in the infected site, enhance its therapeutic effect, and improve patient compliance	China Pharmaceutical University/Wang Wei, Feng Jingwein, Tang Lu	2023
Non-invasive and passive transdermal drug delivery patch for Parkinson’s disease	WO/2023/069778	Development of flexible, non-invasive transdermal patch containing macromolecular drug L-DOPA entrapped within a dissolvable polymer matrix using nanoparticles or nanofibers or thermo-responsive hydrogel, to enable direct and passive drug delivery through the skin	Georgetown University/Charbel Moussa, Makarand Paranjape	2023
Copper sulphide nano-enzyme-antibacterial-peptide-hyaluronic acid composite microneedle as well as preparation method and application of copper sulphide nano-enzyme-antibacterial-peptide-hyaluronic acid composite microneedle	CN115998669	Preparation and application of copper sulphide nano-enzyme and PAF26 antibacterial peptide co-loaded degradable composite (hyaluronic acid and carboxymethyl cellulose) microneedles, as useful carrier system for transdermal drug delivery for improved antifungal effect in the treatment of deep skin fungal infection	Ocean University of China/Zhao Xia, Wang Bingjie, Zhang Wenshang, Tao Jiaojiao, Li Shuang	2023
A nano emulsified phyto-drug for transdermal treatment of diabetes	WO/2023/053077	Formulation and preparation of diosgenin (antidiabetic agent)-loaded oil-in-water nanoemulsion (oil phase: sesame oil and bottle guard seed oil; surfactant: Tween 80; co-surfactant: glycerol) for transdermal drug delivery for treating diabetes	University of South Africa/Oyesolape Basirat Akinsipo, Enock Olugbenga Dare, Samson Oladipupo Oladoyinbo, Lateef Sanni, Deepshikha Katare, T.A.M. Msagati	2023
Self-therapeutic nanoparticle for enhanced topical delivery to skin keratinocytes and treating skin inflammation	US20230048258	Formulation and application method of anti-psoriatic drug composition comprising an alkyl-terminated, PEG-coated gold nanoparticles for transdermal delivery of nanoparticles and effective entry into keratinocytes	The Chinese University of Hong Kong/Chung Hang Jonathan Choi, Ruifang Han, Lok Wai Cola Ho	2023
Pharmaceutical composition of pirfenidone for the management of rheumatoid arthritis	IN202341013877	Preparation of pirfenidone-loaded nanoemulsion and nanoemulsion gel to improve drug skin penetration and therapeutic efficacy for the treatment of rheumatoid arthritis	National Institute of Pharmaceutical Education and Research/Rimsha Nooreen, Shweta Nene, Ganesh B. Vambhurkar, Shashi Bala Singh, Saurabh Srivastava	2023
Biodegradable polymeric compositions, methods of preparation and uses thereof	US20230002566	Design and preparation of nano-sized polymeric compositions comprising polysacharide (starch, alginic acid or hydroxypropyl cellulose) chemically cross-linked by aromatic dialdehyde, in the form of polymeric sheets or particles/capsules, further incorporating lipid, surfactant and/or co-solvent, as potential carriers for bioactive agents for various applications, including topical and transdermal drug delivery	B.G. Negev Technologies and Application Ltd. (Be’er-Sheva, Israel), At Ben-Gurion University/Amnon Sintov	2023
Nano encapsulated knee plaster	IN202221064556	Development of optimized nanofibrous drug eluting scaffold patch loaded with a combination of polymers and drugs as a promising strategy for management of pain and inflammation around articular joint	Pandit Deendayalenergy University/S. Sundar Manoharan, D. Sivaraman, P.S. Pradeep, Sam Scudder, Darshan N Ladva	2022
Slow release microneedle patch and preparation method thereof	CN114668712	Formulation and preparation of sustainable-release microneedle patch containing active nanoparticles composed of metal organic framework dopped with catalase and copper ions and a photosensitizer absorbed on the metal organic framework surface, capable of providing improved photodynamic treatment with reduced toxicity and enhanced anti-tumor effect	Shenzhen College/Huang Peng, He Gang, Li Yashi, Lin Jing	2022
Lipid nanocarrier based transdermal gel for the treatment of pain management	IN202241032969	Development of tapentadol hydrochloride-loaded proniosomal gel as transdermal drug delivery system for pain management, by implementing QbD approach	Sangeetha Govindarajan, Swamislmanickam Mahalingam, Thirumal Valavan Subramanian	2022
Peptide nanoparticles and uses therefor	US20220175634	Development of nanoparticle formulations for non-invasive transdermal delivery of unmodified short peptides	Anterios Inc. (New York, NY, USA)/Jonathan Edelson, Timothy Kotyla	2022
Transdermal drug delivery system and preparation method and application thereof	CN113876743	Formulation, preparation and application of a microneedle patch comprising methotrexate and polydopamine composite manganese dioxide nanoparticles, to provide effective transdermal delivery, reduce methotrexate side effects, improve therapeutic effect and realize imaging diagnosis of rheumatoid arthritis	Shenzhen University/Dong Haifeng, Wu Chaoxiong	2022
Method of using cannabinoids encapsulated in phospholipid carriers for transmucosal and transdermal administration	US20210401766	Preparation and application of cannabinoids (Cannabis sativa derived compounds)-loaded phospholipid vesicles (liposomes) intended for transmucosal and transdermal delivery	NuVessl, Inc. (Calgary, AB, Canada)/Tanya Rhodes, Deborah Duffey	2021
Nanoemulsion system for transdermal delivery of pharmaceutical compositions and other active agents	WO/2021/253049	Preparation of nanoemulsion system for delivery of at least one pharmaceutical or other active agent (drug or cosmetic) through at least an outer skin layer	Sergio Bezerra de Menezes	2021
Transdermal system for synergistic immune-chemotherapy using microneedles and method of treatment thereof	US20210361563	Preparation and application of transdermal system comprising soluble microneedles containing pH-responsive lipid- and polyvinylpyrrolidone-based nanoparticles loaded with anti-PD-1-cisplatin, to facilitate drug release and tumor-targeting	The University of Hong Kong/Yu-xiong Su, Xinmiao Lan, Xi Xie	2021
Bioinspired nanocomposite patch for efficient insulin delivery system	IN202141036011	Preparation of nano chitosan/insulin dermal matrix patches as novel transdermal drug delivery systems to provide improved skin permeation, controlled drug release, and higher patient compliance	S. Khaseel Basha, M. Syed Muzammil, R. Dhandayuthaani, V. Sugantha Kumari	2021
Melanoma chemotherapy drug-loaded nano-liposome and preparation method thereof	CN113244174	Formulation and preparation of chemotherapeutic drug-loaded flexible liposomes modified by combining ceramide grafted hyaluronic acid with sipunculus nudus polypeptide, as effective drug delivery system for targeted subcutaneous therapy of melanoma	Guangdong Laboratory of Southern Ocean Science and Engineering [Zhanjiang]/Ouyang Qianqian, Luo Hui, Huang Yiongmei, Wu Kefeng, Qi Yi, Li Sidong	2021
Preparation method of mesoporous silica nanoparticle transdermal delivery eutectic system	CN113149023	Preparation of mesoporous silica nanoparticles connected with citric acid-amino acid to form eutectic system, providing a new strategy for transdermal drug delivery and potential platform in percutaneous immunotherapy	Kunming University of Science and Technology/Wang Chengxiao, Zhao Zhiyuan, Li Mingjian, Cao Yubiao, Wang Liyun	2021
Siddha poly herbal transdermal pain patch (spot pain patch)-composition and methods of preparation and use thereof for pain management	IN202141013946	Formulation and preparation of poly herbal Siddha medicine-containing transdermal patch for the treatment of pain; the composition can be processed into ointment, dermal spray, nanoemulsion and further applied using iontophoresis, electroporation techniques, microneedles	Central Council for Research in Siddha (Ministry of AYUSH, Govt of India)/K. Kanakavalli, S. Rajalakshmi, K. Samraj, K. Nandhagopal, P. Sathiyarajeswaran, M.S. Shree Devi	2021
Antipsoriatic effects of clobetasol loaded nano structured lipid carriers on imiquimod induced psoriasis	IN202141009486	Development and evaluation of clobetasol-loaded nanostructured lipid carriers incorporated into topical gel (nanogel) for the treatment of psoriasis	Ramesh Reddy Kudamala, Venkata Satyanarayana Suggala, Jayasankar Reddy Veeram, Sucharitha Palagati	2021
Chitosan-pluronic complex and nanocarrier comprising same	US20200222542	Development of polymeric nanocarrier comprising chitosan-pluronic complex and having nano-sponge structure for enhanced delivery of drugs and cosmetics into the skin	Korea Institute of Ceramic Engineering and Technology, Skinmed Co., Ltd. (Daejeon, Republic of Korea)/Won Ii Choi, Sung Hyun Kim, Yong Chul Shin, Jeung Hoon Lee, Jin Hwa Kim, Young Sung Yun	2020

The search string was: [(nano OR nanoparticl*) AND (dermal OR transdermal OR skin) AND delivery] (215 results published from 2020 to 2024, available on PATENTSCOPE database, date of access 10 May 2024).

**Table 6:** Main types of Franz diffusion cells and their properties.

Type	Description	Advantages	Disadvantages
Vertical glass diffusion cell [131] (Original Franz cell)	Cylindrical opening at the bottom, where membrane is mounted and clamped; Donor and receiver compartments separated by glass wall	Simple; Versatile; Widely accepted; Accommodatable to various membranes and formulations	High volume; Static; Labor-intensive; Low throughput
Side-by-side (horizontal) diffusion cell [130,132]	Modified Franz cell with two parallel chambers connected by a narrow slit, for the membrane; Donor and receiver compartments separated by plastic wall	Simulation of in vivo permeation, e.g., gastrointestinal tract (GIT) or blood–brain barrier (BBB)	Complex; Expensive; Less standardized and validated as Franz cell
In-line diffusion cell [130,133]	Flow-type Franz cell with very small receptor volume; Donor and receiver compartments connected by a tubing (membrane placed)	Simulation of dynamic and continuous flow of, e.g., blood or lymph	Difficult to operate and maintain; Less compatible with some membranes and formulations
